# Vascular injury activates the ELK1/SND1/SRF pathway to promote vascular smooth muscle cell proliferative phenotype and neointimal hyperplasia

**DOI:** 10.1007/s00018-023-05095-x

**Published:** 2024-01-27

**Authors:** Chao Su, Mingxia Liu, Xuyang Yao, Wei Hao, Jinzheng Ma, Yuanyuan Ren, Xingjie Gao, Lingbiao Xin, Lin Ge, Ying Yu, Minxin Wei, Jie Yang

**Affiliations:** 1 Division of Cardiovascular Surgery, Cardiac and Vascular Center, The University of Hong Kong-Shenzhen Hospital, Shenzhen, China; 2 Department of Biochemistry and Molecular Biology, Department of Immunology, School of Basic Medical Science, Tianjin Medical University, Tianjin, China; 3 Key Laboratory of Immune Microenvironment and Disease (Ministry of Education), and Key Laboratory of Cellular and Molecular Immunology, Tianjin, China; 4 The Province and Ministry Co-Sponsored Collaborative Innovation Center for Medical Epigenetics, Tianjin Medical University, Tianjin, China; 5 State Key Laboratory of Experimental Hematology, Tianjin, China; 6 Eye Institute & School of Optometry and Ophthalmology, Tianjin Medical University Eye Hospital, Tianjin, China

**Keywords:** VSMC phenotype switching, Neointimal hyperplasia, SND1, ELK1, SRF

## Abstract

**Background:**

Vascular smooth muscle cell (VSMC) proliferation is the leading cause of vascular stenosis or restenosis. Therefore, investigating the molecular mechanisms and pivotal regulators of the proliferative VSMC phenotype is imperative for precisely preventing neointimal hyperplasia in vascular disease.

**Methods:**

Wire-induced vascular injury and aortic culture models were used to detect the expression of staphylococcal nuclease domain-containing protein 1 (SND1). SMC-specific *Snd1* knockout mice were used to assess the potential roles of SND1 after vascular injury. Primary VSMCs were cultured to evaluate SND1 function on VSMC phenotype switching, as well as to investigate the mechanism by which SND1 regulates the VSMC proliferative phenotype.

**Results:**

Phenotype-switched proliferative VSMCs exhibited higher SND1 protein expression compared to the differentiated VSMCs. This result was replicated in primary VSMCs treated with platelet-derived growth factor (PDGF). In the injury model, specific knockout of *Snd1* in mouse VSMCs reduced neointimal hyperplasia. We then revealed that ETS transcription factor ELK1 (ELK1) exhibited upregulation and activation in proliferative VSMCs, and acted as a novel transcription factor to induce the gene transcriptional activation of *Snd1.* Subsequently, the upregulated SND1 is associated with serum response factor (SRF) by competing with myocardin (MYOCD). As a co-activator of SRF, SND1 recruited the lysine acetyltransferase 2B (KAT2B) to the promoter regions leading to the histone acetylation, consequently promoted SRF to recognize the specific CArG motif, and enhanced the proliferation- and migration-related gene transcriptional activation.

**Conclusions:**

The present study identifies ELK1/SND1/SRF as a novel pathway in promoting the proliferative VSMC phenotype and neointimal hyperplasia in vascular injury, predisposing the vessels to pathological remodeling. This provides a potential therapeutic target for vascular stenosis.

**Graphical abstract:**

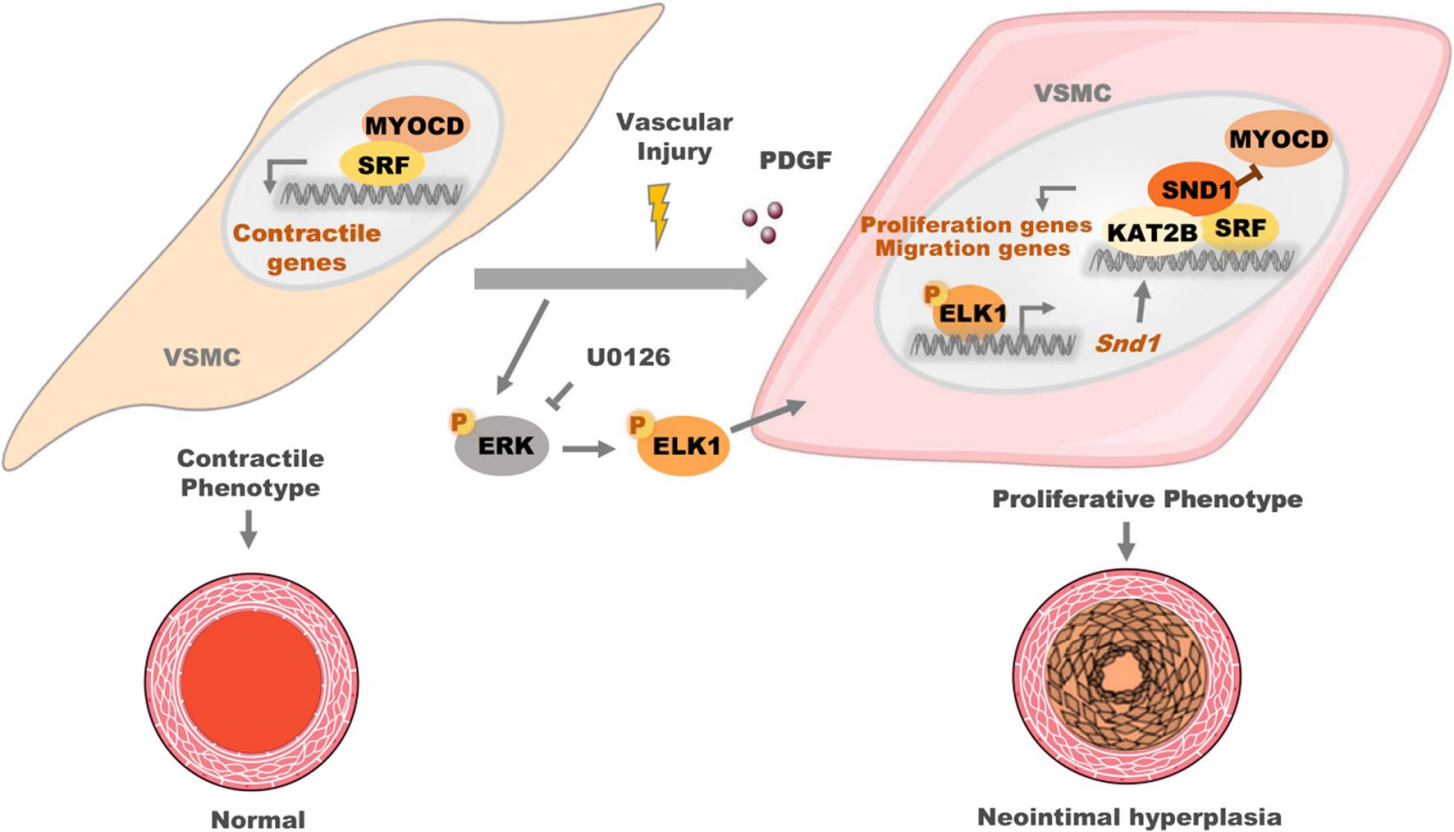

**Supplementary Information:**

The online version contains supplementary material available at 10.1007/s00018-023-05095-x.

## Introduction

Vascular stenosis is one of the most crucial pathophysiological processes in vascular disease, while restenosis is the major limitation of percutaneous coronary intervention or bypass grafting [1, 2]. Vascular smooth muscle cells (VSMCs), which are the main components of the vascular wall, are critically involved in the processes of vascular stenosis or restenosis [3]. The VSMCs exhibit highly plastic and dynamic ability to switch from high differentiation to proliferation, migrate from the tunica media into the intima, and secrete extracellular matrix to form the irreversible neointima, thus playing a critical role in most vascular disease [4, 5]. Therefore, investigation of the molecular mechanisms and pivotal regulatory factors of the proliferative VSMC phenotype is imperative for the precise prevention of vascular stenosis or restenosis.

Distinct VSMC phenotypes express specific marker proteins that have different functions. Under normal conditions, differentiated VSMCs express high levels of contractile proteins that regulate vascular tone and control blood pressure [6]. VSMCs dedifferentiate and express low levels of contractile proteins while expressing high levels of proliferative and migratory proteins in pathological conditions, contributing to neointimal hyperplasia and vascular stenosis [6]. Significant progress has been made in understanding the molecular mechanisms that regulate VSMC phenotypes. For example, the Rho-actin signaling pathway induces the transcription factor serum response factor (SRF) to bind to myocardin (MYOCD) and modulates the expression of genes encoding contractile proteins [7, 8]. Ras-extracellular signal-regulated kinase (Ras-ERK) signaling activates cell cycle-related gene transcription by inducing interaction between SRF and E26 transformation-specific (ETS) transcription factors ELK1, ELK3, and ELK4 [9]. However, the molecular mechanisms underlying the phenotype switching of quiescent VSMCs to a proliferative phenotype are still poorly understood.

Staphylococcal nuclease domain-containing protein 1 (SND1), also known as p100 or Tudor staphylococcal nuclease (Tudor-SN), is a multifunctional protein [10]. It is highly expressed in proliferating cells and tissues [11]. However, it is barely expressed in differentiated or quiescent cells [12]. In this study, we found that vascular injury markedly upregulated SND1 expression in VSMCs. We developed SMC-specific *Snd1* knockout (*Snd1-F/F Tagln-Cre*) mice; although the vasculature was phenotypically indistinguishable from *Snd1-F/F* mice, the *Snd1-F/F Tagln-Cre* mice developed substantially less neointima after wire-injury. This suggests that deficiency of SND1 in VSMC inhibits VSMC dedifferentiation and decreases VSMC proliferation in injured vessels. Thus, SND1 is likely to play an important role in promoting the proliferative VSMC phenotype, making it a potential therapeutic target for vascular restenosis.

## Materials and methods

### Animal studies

All Sprague–Dawley (SD) rats (RRID: RGD 70508) and C57BL/6 J (RRID: IMSR_JAX: 000664) mice were housed under a 12 h light–dark cycle, with free access to water and the standard laboratory diet (crude protein ≥ 20%, crude fat ≥ 4%, Beijing Keao Xieli Feed Co.,Ltd, #1016706476803973120). The animals were randomly allocated to the experimental groups. Sex is a considerable biological variable [13], and it has been reported that estrogens exert an inhibitory effect on neointimal formation in carotid artery injury models [14, 15]. Therefore, to avoid the influence of estrogen in female animals, only male mice and rats were used in the artery wire-injury model.

The *Snd1-F/F* mice have been reported in our previous study [16]. The *Tagln-Cre* mice (RRID:IMSR_JAX:017491) were shared by Y. Yu lab from Tianjin Medical University [17]. The SMC-specific *Snd1* knockout (*Snd1-F/F Tagln-Cre*) mouse strain was generated by crossing *Snd1-F/F* mouse with *Tagln-Cre* transgenic mouse. The targeted deletion of *Snd1* in VSMCs was verified by polymerase chain reaction (PCR). The primers of genotyping are shown in the Supplementary Table S1. The protein levels of SND1 in VSMCs and other tissues were detected by western blotting and immunohistochemistry.

### Isolation of thoracic aorta and primary VSMCs

8–12-week-old male mice were euthanized, and the aortas were dissected from the aortic arch to the iliac bifurcation, and the adventitia and endothelial cell layers were removed. The isolated aortic tissues were either directly harvested for protein and RNA extraction, or cut into 3–5 mm long pieces and cultured in Dulbecco’s modified Eagle’s medium (DMEM) (Biological Industries, 06-1055-57-1ACS) containing 20% fetal bovine serum (FBS) (Biological Industries, 04-001-1ACS) and 1% penicillin/streptomycin-glutamine at 37 °C, 5% CO_2_. Cells from the cultured aorta in passages 3–5 were used in all experiments.

### Construction of lentivirus plasmids

To create the shElk1-1 and shElk1-2 plasmids, shRNAs targeting *Elk1* were cloned into a TRC2-pLKO-Puro-Vector (Sigma-Aldrich, SHC201) with AgeI and EcoRI sites. The sequences of shRNA were designed using “Invitrogen Block-iT RNAi Designer (thermofisher.com)” (Supplementary Table S2). The cDNA sequences of *Elk1* (NM_007922.5) and *Snd1* (NM_019776.2) were cloned into the pLVX-IRES-Puro-Vector (Clontech, 632183) containing a FLAG tag. The wild type and ELK1 binding sites (–CCGGAAGT–) mutant promoter sequences of *Snd1* were cloned into the Gluc-ON™ promoter-reporter vector (GeneCopoeia, EZX-LvPG04). HEK293T cells (RRID:CVCL_1926) were co-transfected with 11.25 μg of the above expression plasmids, 3.95 μg of pMD2.G (RRID: Addgene_12259) packaging plasmid, and 7.3 μg of psPAX2 (RRID: Addgene_12260) envelope plasmid using a polyethyleneimine transfection reagent. The medium was replaced with fresh DMEM containing 10% FBS after 6–8 h of transfection. The viral supernatant was collected and filtered through a 0.45 μm filter at 24 and 48 h after transfection.

### Rat carotid artery wire injury model

Male rats weighing 200–230 g were anesthetized for 3 min with 3–4% isoflurane in oxygen, and the maintenance dose during the operation was 2% isoflurane in oxygen. To prevent thrombosis, 0.1% heparin sodium was injected into the tail vein at 0.1 mg/100 g body weight. The rat necks were disinfected with 75% alcohol, and the hairs were depilated. Following the isolation of the common carotid artery via a midline cervical incision, a guidewire (approximately 0.8 mm) was inserted into the left external artery and advanced back and forth approximately 3 cm proximally. This step was repeated approximately five times and the wire was carefully removed. The right carotid artery of the sham group was exposed without injury. After the surgery, the opening was sutured with a 4–0 nylon suture and disinfected with an iodophor. The rats were placed on a heating blanket until they recovered from anesthesia. The carotid arteries were collected after 14 days of injury for subsequent experiments.

### Mouse femoral artery wire injury model

Male mice in 8–12-week-old were anesthetized for 1 min with 2% isoflurane in oxygen, and the maintenance dose during the operation was 1% isoflurane in oxygen. After disinfection and depilation in the left lower limbs of the mice, the left femoral artery was isolated by blunt dissection under a surgical microscope. The distal artery was looped with a 6–0 nylon suture, and a 0.38 mm guide wire (Cook Inc, Bloomington, IN) was inserted into the arterial lumen through an arteriotomy in the distal perforating branch. The wire was left in the artery for 2 min to denude the artery and was carefully removed. The right femoral artery was used as the sham group. The nylon suture was released to restore the blood flow, and the opening was sutured with a 6–0 nylon suture. The mice were placed on a heating blanket until recovery, and the femoral arteries were collected after 28 days.

The mouse femoral artery injury model was also performed with or without the U0126 treatment. U0126 was purchased from Selleck (S1102) and dissolved in dimethyl sulfoxide (DMSO) to a concentration of 0.1 mg/mL. It was injected intraperitoneally into mice every 3 days (1 mg/kg) after wire injury. The dose of U0126 was determined based on a previous study [18]. The control group was injected with a similar volume of DMSO.

### Immunohistochemistry

Rat or mouse artery tissues were fixed in 4% paraformaldehyde for 24 h and embedded in paraffin. The tissues were then deparaffinized, rehydrated, and subjected to antigen retrieval by heating for 20 min in a microwave oven. Endogenous peroxidase activity was quenched for 10 min using peroxide blockers (ZSGB-BIO, PV-9000). The sections were blocked in 5% bovine serum albumin in PBS for 30 min at room temperature before being incubated with specific primary antibodies overnight at 4 °C, then incubated with secondary antibody for 30 min at room temperature. Next, the sections were stained by diaminobenzidine (ZSGB-BIO, ZLI-9018), and counterstained with hematoxylin. Images were captured using the inverted microscope. The detailed information of the antibodies is shown in Supplementary Table S6.

### Cell counting kit-8 (CCK-8) assay

Primary VSMCs which were isolated from *Snd1-F/F* and *Snd1-F/F Tagln-Cre* mice, or the cultured VSMCs infected with lentiviral plasmids, were seeded in the 96-well plates (5 × 10^3^ cells per well) overnight. The VSMC proliferation ability was detected using the CCK-8 cell proliferation assay kit (DoJindo, CK04) according to the manufacturer’s instructions. Absorbance was detected at 450 nm. Continuous testing was performed for 6 days.

### Wound healing assay

The primary VSMCs were seeded in 6-well plates (3 × 10^5^ cells per well). Three linear wounds were gently introduced into the cell monolayer through a 20 μL tip, and fresh medium containing 1% FBS was added. Images were collected at 0 and 36 h after scratching. The gap distance covered was evaluated using the ImageJ (2 ×) software (RRID:SCR_003070).

### Live-cell imaging assay

The primary VSMCs were seeded in 12-well plates (20 μL per well). Cells were imaged for 4 h intervals at 37 °C with 5% CO_2_ in air. Images were taken every 20 min using an inverted microscope and analyzed with ImageJ (2 ×) software. Greater than 30 cells were analyzed for each experiment and sample and results provided are from three independent experiments.

### Collagen-gel contraction assay

A collagen gel solution was prepared using 1 mL of type I collagen (CORNING, 354236) at a concentration of 2.4 mg/mL in 1 × PBS buffer (pH 7.0). VSMCs were seeded in 12-well plates containing collagen gels (2 × 10^4^ cells per well). The contraction of the gel was photographed at 36 h, and the gel size was calculated using ImageJ (2 ×) software. The extent of collagen gel contraction was measured by dividing the gel size at 36 h by the initial gel size (% Contraction = Gel Area (cm^2^)/Well Area (cm^2^) × 100%).

### 5-ethynyl-2′-deoxyuridine (EdU) assay

VSMCs were seeded in 48-well plates (1 × 10^4^ cells per well). The next day, cells were incubated with EdU reagent (1 μM) (Beyotime, C0071S) for 2 h at 37 °C. Then cells were fixed in 4% PFA/PBS for 10 min and permeabilized with 0.3% TritonX-100 for 10 min. After washing with PBS (3 × 3 min), cells were incubated in EdU Click-iT reaction buffer (10 mM sodium-L-ascorbate, 5 μM Alexa Fluor 488-azide, and 2 mM CuSO_4_) for 30 min in the dark. Nuclei were visualized using DAPI, and images were captured using a confocal laser scanning microscope. The proliferation rate was assessed by positive cell number/total cell number.

### Western blotting analysis

Harvested VSMCs or frozen tissues were washed with cold 1 × PBS and lysed with Cell Lysis Buffer (Solarbio, R0010) containing protease inhibitor cocktail (Roche Applied Science, 04693132001). Protein concentration was determined by bicinchoninic acid assay (Thermo Fisher Scientific, 23227), and samples were boiled with 1 × loading buffer. Samples were run in SDS-PAGE gel in 1 × Tris–Glycine-SDS running buffer, and the concentration of the SDS-PAGE gel depended on the molecular weight of the interesting proteins. Proteins were transferred to polyvinylidene difluoride membranes (Merck Millipore, IPVH00010). Membranes were blocked in 5% BSA and incubated with indicated antibodies overnight at 4 °C (Supplementary Table S6), followed by incubation with appropriate secondary antibodies. Blots were visualized using chemiluminescence reagents (Thermo Scientific, 32132), ACTB was used as an internal control, and the results were quantified using ImageJ (2 ×) software.

### Real-time quantitative polymerase chain reaction (RT-qPCR)

Total RNA was extracted from cells or vascular tissues using TRIzol reagent (Vazyme, R401) according to the manufacturer’s instructions and then reverse transcribed to cDNA using reverse transcription reagent kits (Thermo Fisher Scientific, K1622). Real-time PCR was performed using SYBR Green mix (Vazyme, Q511-02). Each sample was analyzed in triplicate, and the results were normalized to *Actb*. (The primer sequences are shown in Supplementary Table S3).

### Luciferase activity assay

VSMCs were seeded 2 × 10^5^ per well in 6-well plates. The next day, cells were infected with the empty GLuc-Vector (GeneCopoeia, EZX-LvPG04) or GLuc-promoter reporter plasmids, respectively. After different stimulation, 0.1 mL of the cell culture medium of each group was collected. Luciferase activities were measured using a luciferase assay kit (GeneCopoeia, LF031). In brief, the secreted Alkaline Phosphatase (SEAP) or GLuc assay working solution was prepared by adding 10 μL of substrate GL to 1 mL of 1 × AP Buffer or GL-S and mixed well, then incubated at room temperature. The culture medium samples were added into a 96-well plate (10 μL per well), then the working solutions were added to the samples (100 μL per well), and incubated at room temperature for 1 min. The GLuc and SEAP activity signals were measured by the luminometer. The GLuc activities were normalized using the SEAP signal as a standard internal control (ratio of GLuc and SEAP activities).

### Chromatin immunoprecipitation assay (ChIP)

High-power sonication for 20 cycles of 10 s ON and 10 s OFF, 60% of the pulse, was used to generate chromatin fragments of 300–1000 bps from cell nuclear lysates. Magna ChIP Protein A + G Magnetic Beads (2–5 μg) (Millipore, 16–663) were incubated with the indicated antibody, IgG as the negative control. The complex was then incubated with 100 μg of cell nuclear lysate with gentle rotation. The purified immunoprecipitated DNA samples were collected with a QIA quick Purification Kit (Qiagen, 28104) and detected by qPCR with specific primers (Supplementary Table S4).

### Co-immunoprecipitation (Co-IP) assay

Total cell lysates were extracted using cell lysis buffer (50 mM Tris–HCl (pH 8.0), 150 mM NaCl, 0.2% Nonidet P-40, and 2 mM EDTA) supplemented with protease inhibitor cocktails. Pierce Protein A/G agarose beads (30 µL) (Thermo Fisher Scientific, 20422) were incubated with indicated antibody or IgG as a negative control for 6–8 h at 4 °C with constant rotation. Then, 2 mg of total cell lysate was added, and the incubation was continued for an additional 12 h. 1% of the total cell lysate was used as the input. The beads were washed five times with PBS containing 0.1% Tween-20 the next day. The precipitated proteins were eluted from the beads by resuspending them in 2.5 × loading buffer and boiling them for 10 min. The bound proteins were analyzed using 7.5% SDS-PAGE and blotted with the indicated antibodies.

### Silver staining and mass spectrometry

SND1 antibody was incubated with 30 µL Pierce Protein A/G agarose beads (Thermo Fisher Scientific, 20,422) for 6–8 h at 4 °C with constant rotation, IgG as the negative control. Then, 2 mg of total cell lysate was added, and the incubation was continued for an additional 12 h. The beads were washed with cold 1 × PBS and 0.1% Tween-20, and visualized by silver staining on 8% SDS-PAGE using the Pierce silver stain kit (Thermo Fisher Scientific, 24600). Distinct protein bands on the gel were excised and subjected to in-gel tryptic digestion. The resulting peptides were separated using reverse-phase liquid chromatography on an easy-nLC 1000 system (Thermo Fisher Scientific) and directly sprayed into a Q Exactive Plus mass spectrometer (Thermo Fisher Scientific). Mass spectrometry analysis was carried out in data-dependent mode with an automatic switch between a full MS and an MS/MS scan in orbitrap high-resolution mass spectrometry. All MS/MS spectra were searched against the Uniport-Mice protein sequence database by using the PD search engine (Thermo Fisher Scientific, v 2.1.0) with an overall false discovery rate for peptides of less than 1%.

### Recombinant protein purification

The GST-vector, GST-SND1, GST-SRF, and their truncated fusion proteins were expressed in BL21 Escherichia coli (E. coli) and purified by the Pierce Glutathione Agarose (Thermo Fisher, 16100). The recombinant protein SRF was expressed in rabbit reticulocyte lysates (Promega, L1170). His-SND1 was produced in BL21(DE3) E. coli and purified using His-Select nickel affinity gel (Sigma-Aldrich, P6611).

### Electrophoretic mobility shift assay (EMSA)

The biotin-labeled and unlabeled probes were synthesized by GENEWIZ company (Supplementary Table S5). EMSA experiments were performed using the chemiluminescent EMSA kit (Beyotime, GS009) according to the manufacturer’s protocol. Biotin-labeled probes (20 fmoL) were incubated with 0.1 μg SND1- or SRF-purified proteins for 10 min at room temperature in the binding buffer. For competition experiments, 200-fold excess of unlabeled or nonspecific probes were preincubated with purified proteins for 20 min before adding the biotin-labeled probes. Reaction mixtures were resolved by 6% polyacrylamide gel pre-electrophoresed for 30 min in 0.5 × tris borate/EDTA and electrophoresed at 100 V before being transferred to positively charged nylon membranes (Beyotime, FFN10). The transferred DNA molecules were cross-linked to the membrane at 120 mJ cm^−2^ and detected using horseradish peroxidase-conjugated streptavidin.

### Statistical analysis

Statistical analysis was performed using GraphPad Prism software (RRID: SCR_002798). Data from multiple biological experiments are presented as the mean ± SD. The unpaired two-tailed Student’s t-test was used to compare two groups of data. The one or two or three-way analysis of variance (ANOVA) was used to compare multiple groups of data. The repeated measures ANOVA was used to compare different time changes data. The ANOVA was followed by Bonferroni multiple-comparison post-hoc correction. *P* < 0.05 was considered statistically significant.

## Results

### SND1 expression is upregulated in proliferative VSMCs under pathological conditions

**Fig. 1:**
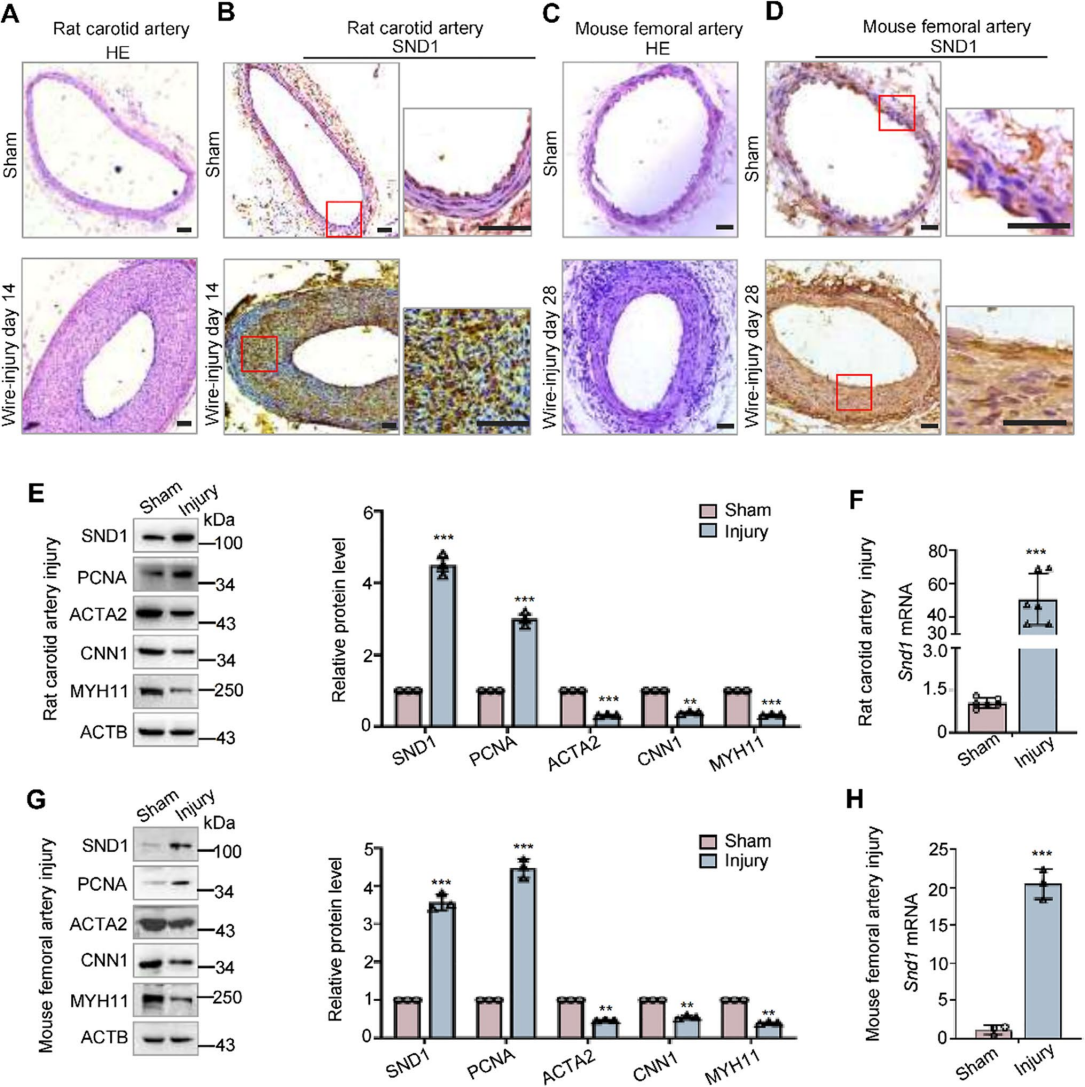
Vascular injury increased the expression of SND1. Wire-induced rat carotid artery injury (*n* = 6 /group) and mouse femoral artery injury (*n* = 6 /group) models were generated. The carotid and femoral arteries were collected at 14 or 28 days after the injury, respectively. **A**, **C** Representative HE staining of carotid or femoral arteries from sham-operated and wire-injured groups. **B**, **D** Immunohistochemical staining of SND1 in carotid or femoral arteries from sham-operated and wire-injured groups. **E**, **G** The protein levels of SND1, PCNA, ACTA2, CNN1, MYH11, and ACTB were detected by western blotting. All western blotting results were analyzed by ImageJ (2 ×) software. ** F, H** The mRNA level of *Snd1* was detected by RT-qPCR. Scale bar, 200 μm in A and B; 100 μm in C and D. Data are presented as the mean ± SD. Statistical analysis was performed by unpaired two-tailed Student’s *t*-test. **P < 0.01; ***P < 0.001

Previously, we reported that SND1 was highly expressed in proliferating cells, but barely expressed in differentiated or quiescent cells [11, 12]. To our knowledge, VSMCs have a highly plastic ability to switch from a differentiated contractile state to a proliferative and migratory phenotype in vascular diseases or restenosis [3–6]. Therefore, we generated wire-induced rat carotid artery injury and mouse femoral artery injury models to imitate the implanted stent injury and detected the expression pattern of SND1 in VSMC phenotype switching. Hematoxylin-eosin (HE) staining revealed severe intimal hyperplasia in both rat carotid artery (Fig. 1A) and mouse femoral artery (Fig. 1C) induced by wire-injury and immunohistochemical staining demonstrated that the expression of SND1 was significantly upregulated in the neointima compared with sham-operated rat carotid or mouse femoral artery (Fig. 1B and D). The VSMC marker α-smooth muscle actin (ACTA2) was used to confirm that the main components of the neointima were VSMCs as previous report [19] (Supplementary Fig. S1, A and B), and the normal rabbit IgG served as the negative control (Supplementary Fig. S1, C and D). Meanwhile, western blotting analysis further indicated an increased expression of SND1 in the injured arteries compared with the sham-operated arteries (Fig. 1E and G), and the mRNA level of *Snd1* was also upregulated in the injured arteries (Fig. 1F and H). The increased levels of proliferating cell nuclear antigen (PCNA) and decreased levels of ACTA2, calponin (CNN1), and smooth muscle myosin heavy chain (MYH11) in the injured arteries illustrated the switch of VSMCs from a contractile state to a proliferative phenotype (Fig. 1E and G).

**Fig. 2:**
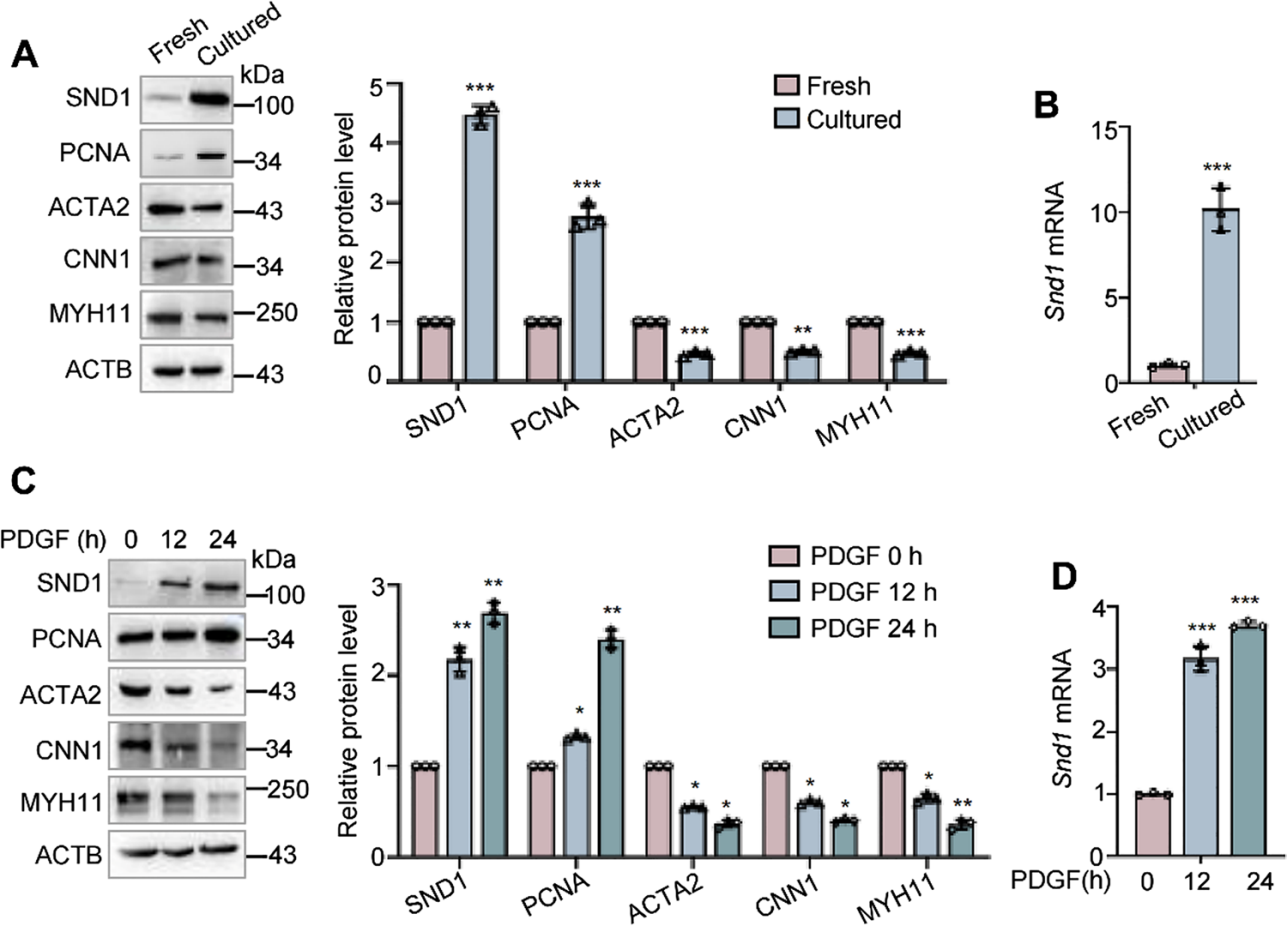
The expression of SND1 increased in proliferating VSMCs in vitro. **A** Mice thoracic aortas were isolated and collected (Fresh) or cultured for 3 days. The protein levels of SND1, PCNA, ACTA2, CNN1, and MYH11 in the fresh and cultured aortas were detected by western blotting, and **B** the mRNA level of *Snd1* was detected by RT-qPCR. **C** The primary VSMCs were cultured and seed into a 6 cm dish, cells were allowed to grow until the cells were 70 to 80% confluent and treated with PDGF (40 ng/mL) for 24 h after being cultured in serum-free DMEM for 24 h, 2 × 10^6^ cells were harvested. The protein levels of SND1, PCNA, ACTA2, CNN1, MYH11, and ACTB were detected by western blotting, and **D** the mRNA level of *Snd1* were detected by RT-qPCR. All western blotting results were analyzed by ImageJ (2 ×) software. Data are presented as the mean ± SD (*n* = 3). Results in A and B were analyzed by unpaired two-tailed Student’s *t*-test, and results in C and D were analyzed by one-way ANOVA followed by Bonferroni post-hoc correction. *P < 0.05; **P < 0.01; ***P < 0.001

Furthermore, aortic tissues were directly collected for total protein and RNA extraction (fresh aorta), or cut into pieces using surgical scissors, and cultured in DMEM containing 20% fetal bovine serum (FBS) for 3 days (cultured aorta). Stimulation by mechanical shear force or cytokines in the FBS is similar to vascular injury in vivo. Consistent with the wire-injured artery, the protein level of SND1 was upregulated in the cultured aorta, as well as the proliferative marker PCNA, compared to the fresh aorta (Fig. 2A). However, protein levels of ACTA2, CNN1, and MYH11 were downregulated in the cultured aorta (Fig. 2A). The mRNA level of *Snd1* was also upregulated in cultured aortas (Fig. 2B). These data further indicated that the transition of VSMC from a contractile state to proliferation is correlated with the enhanced expression of the SND1 protein.

Platelet-derived growth factor (PDGF) plays an essential role in the switch of VSMC from the contractile to proliferative phenotype in vascular injury [20]. To investigate whether PDGF could regulate the expression of SND1, primary VSMCs were cultured in serum-free DMEM for 24 h, then stimulated with PDGF (40 ng/mL) for 12 or 24 h, and harvested for total protein and RNA extraction. We found that both protein and mRNA levels of SND1 increased in response to PDGF stimulation in a time-dependent manner (Fig. 2C and D). The correlation between enhanced SND1 expression and VSMC phenotype switching implies that SND1 may be involved in neointimal hyperplasia caused by vascular injury.

### Deficiency of SND1 in VSMCs inhibits injury-induced neointima hyperplasia

We constructed SMC-specific *Snd1* knockout (*Snd1-F/F Tagln-Cre*) mice to assess the potential role of SND1 in wire injury-induced neointimal hyperplasia in vivo. The result of genotyping is shown in Supplementary Fig. S3A. For the SND1 was barely expressed in the mature differentiated VSMCs, we detected the expression of SND1 in the thoracic aortas of 7-day-old (7 d) mice. The results showed that the SND1 was nearly undetectable in the thoracic aortas of the 7 d *Snd1-F/F Tagln-Cre* mice (Supplementary Fig. S3, B and D). Meanwhile, the results showed that SND1 was not deleted in the liver, kidney, and spleen tissues of *Snd1-F/F Tagln-Cre* mice (Supplementary Fig. S3, C and D). Considering that the *Tagln-Cre* is active in the heart during development [21], the conditional deletion of *Snd1* may also occur in early cardiomyocytes. However, no differences in morphology (Supplementary Fig. S3E) were observed in the heart of adult *Snd1-F/F Tagln-Cre* mice comparing with *Snd1-F/F* mice. Moreover, the deletion of *Snd1* did not influence the life span of mice. These results indicated that a workable system of mouse with the SMC-*Snd1* knockout was constructed.

**Fig. 3:**
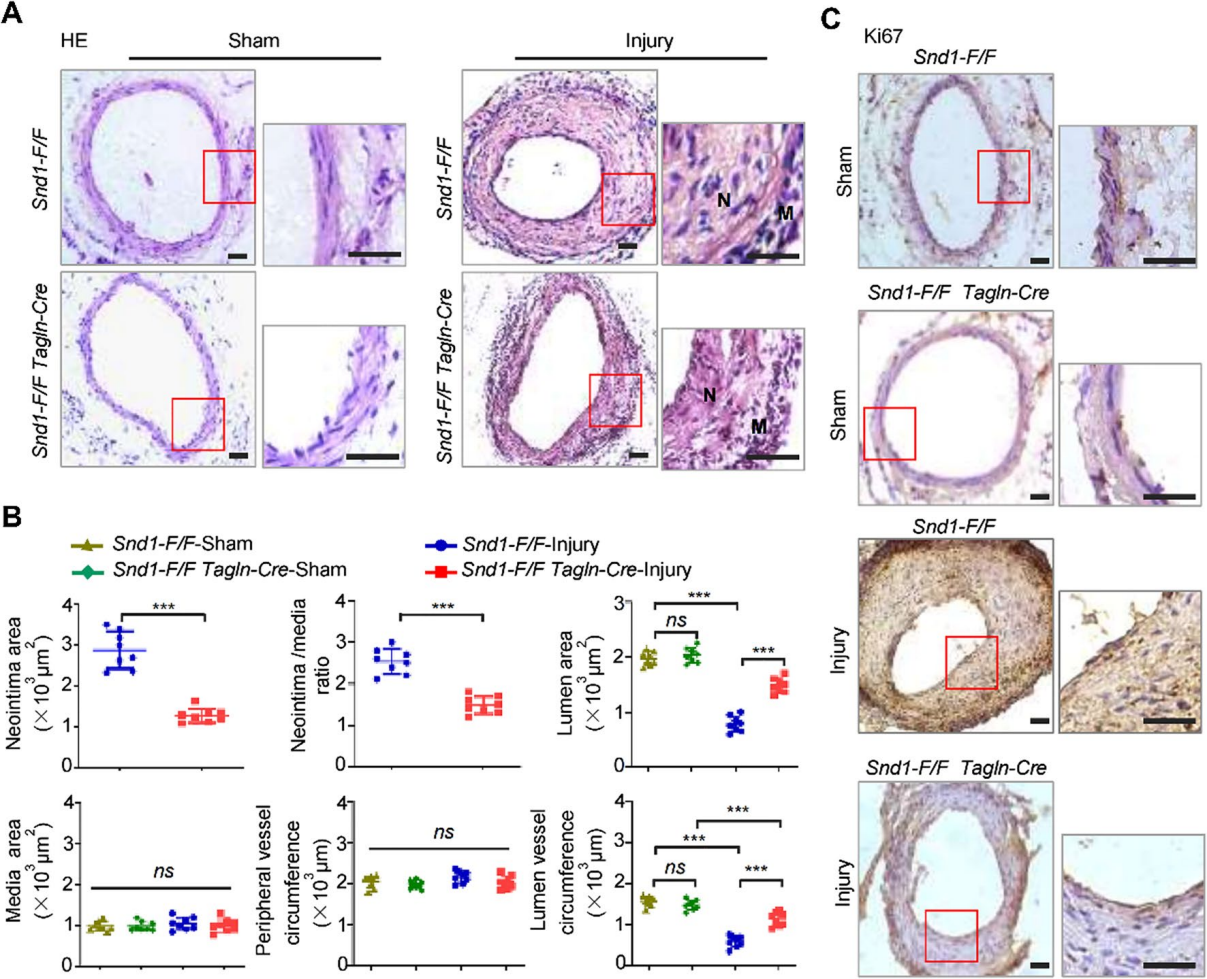
The neointimal hyperplasia was inhibited in the *Snd1-F/F Tagln-Cre* mice after wire-induced vascular injury. Wire-induced mouse femoral artery injury models were generated. The femoral arteries were collected 28 days after the injury (*n* = 8 /group). **A** Representative HE staining of sham-operated and wire-injured femoral arteries from *Snd1-F/F* and *Snd1-F/F Tagln-Cre* mice. (*N* neointima, *M* media). **B** Averaged data of the neointima area, neointima/media ratio, lumen area, media area, peripheral vessel circumference, lumen vessel circumference of sham-operated or wire-injured femoral arteries (*n* = 8 /group). **C** Immunohistochemical staining of Ki67 (*n* = 3 /group). Scale bar, 100 μm. Data are presented as the mean ± SD. Results were analyzed by unpaired two-tailed Student’s *t*-test and two-way ANOVA followed by Bonferroni post-hoc correction. ***P < 0.001; *ns* no significance

The wire-induced mouse femoral artery injury model was constructed to detect the function of SND1 in neointimal hyperplasia. There was no obvious difference in the vascular morphology of the femoral artery between *Snd1-F/F Tagln-Cre* and *Snd1-F/F* mice in the sham group (Fig. 3A), as shown by the similar lumen area, media area, peripheral vessel circumference, and lumen vessel circumference (Fig. 3B). However, the wire injury-induced pronounced neointimal hyperplasia in the *Snd1-F/F* mice was inhibited in the *Snd1-F/F Tagln-Cre* mice (Fig. 3A), as evidenced by decreased neointima area and neointima/media ratio, increased lumen area and the lumen vessel circumference (Fig. 3B). Of note, the media area experienced no significant change, illustrating that vascular stenosis was mainly determined by intimal hyperplasia. Supportively, the immunohistochemical staining of Ki67 was weakened in the samples of wire-injured *Snd1-F/F Tagln-Cre* mice compared with *Snd1-F/F* mice (Fig. 3C). These indicate that knockout of SND1 remarkably suppresses wire-injury-induced VSMC proliferation and neointima hyperplasia.

### SND1 promotes VSMC proliferative phenotype

**Fig. 4:**
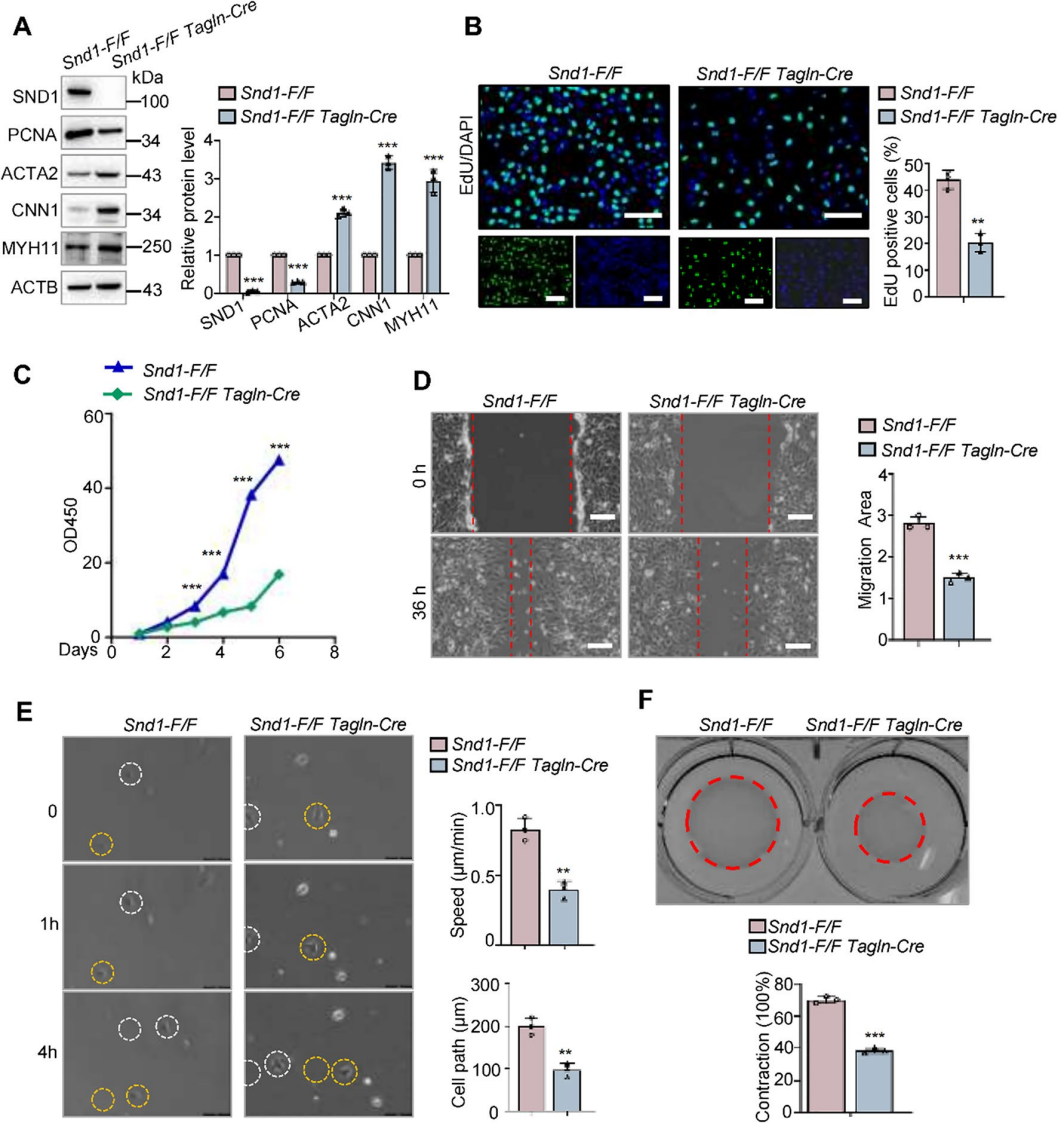
Knockout of *Snd1* prevented VSMC proliferative phenotype in vitro. **A** Primary VSMCs from *Snd1-F/F* and *Snd1-F/F Tagln-Cre* mice were isolated and cultured. The protein levels of SND1, PCNA, ACTA2, CNN1, MYH11, and ACTB were detected by western blotting. All western blotting results were analyzed by ImageJ (2 ×) software. Cell proliferation ability of the primary VSMCs from *Snd1-F/F* and *Snd1-F/F Tagln-Cre* mice was detected by **B** EdU incorporation (1 × 10^4^ cells in per 48 well plates) and **C** CCK-8 assay (5 × 10^3^ cells in per 96-well plates). **D**, **E** Cell migration ability was detected by wound-healing assays (3 × 10^5^ cells in per 6-well plates) and living cell image (1 × 10^3^ cells in per 12-well plates). **F** Cell contraction ability was detected by collagen gel contraction assay (2 × 10^4^ cells in per 12-well plates). The extent of collagen gel contraction (% Contraction) = Gel Area (cm^2^) /Well Area (cm^2^) × 100%. Scale bar, 100 μm. Data are presented as the mean ± SD (*n* = 3). Results in A, B, D, E, and F were analyzed by unpaired two-tailed Student’s *t*-test, results in C were analyzed by repeated measures ANOVA followed by Bonferroni post-hoc correction. **P < 0.01; ***P < 0.001

We have observed that SND1 deficiency could suppress neointimal hyperplasia. To further investigate whether SND1 is involved in the phenotype switching of VSMC, we examined the potential effect of SND1 on VSMC proliferation, migration, and contraction ability. Primary VSMCs were isolated from *Snd1-F/F* and *Snd1-F/F Tagln-Cre* mice. The knockout efficiency of the SND1 protein in primary VSMCs was detected (Fig. 4A). The decreased PCNA and increased ACTA2, CNN1, and MYH11 levels in the *Snd1-F/F Tagln-Cre* VSMCs illustrated the contractile phenotype of VSMCs (Fig. 4A). The 5-ethynyl-2′-deoxyuridine (EdU) incorporation (Fig. 4B) and cell counting kit-8 (CCK-8) assay (Fig. 4C) revealed that knockout of *Snd1* in the *Snd1-F/F Tagln-Cre* VSMCs significantly diminished the primary VSMC proliferation ability compared with *Snd1-F/F* VSMCs. Knockout of *Snd1* reduced the migration ability of VSMCs, as evaluated by the wound-healing assay (Fig. 4D). To rule out any influence of proliferation on cell migration in our analyses, we performed live-cell imaging to track the migration of individual cells. The result showed reduced cell motility in *Snd1-F/F Tagln-Cre* VSMCs compared to the *Snd1-F/F* VSMCs (Fig. 4E). The results of the collagen contraction assay showed that the size of the collagen gel matrix was smaller in the primary *Snd1-F/F Tagln-Cre* VSMCs than in the *Snd1-F/F* VSMCs (Fig. 4F). It indicated that knockout of *Snd1* increased the contraction ability of VSMCs.

In addition, the SND1 protein was ectopically overexpressed in primary VSMCs by infecting with pLVX-IRES-FLAG-SND1 lentivirus. Western blotting analysis showed that overexpression of the SND1 protein promoted the VSMC proliferative phenotype, as the expression of PCNA was increased, and ACTA2, CNN1, and MYH11 were decreased (Supplementary Fig. S4A). EdU incorporation (Supplementary Fig. S4B) and CCK-8 assay (Supplementary Fig. S4C) further confirmed that overexpression of SND1 significantly accelerated the proliferation of VSMCs. Moreover, the wound healing assay showed that SND1 overexpression enhanced the migration ability of VSMCs (Supplementary Fig. S4D). However, the contraction ability of the VSMCs was reduced with overexpression of SND1, as tested by the functional collagen contraction assay (Supplementary Fig. S4E). These results indicate that SND1 promotes VSMC proliferative phenotype.

### SND1 competes with MYOCD to interact with SRF and promotes the proliferation- and migration-related gene transcriptional activation

**Fig. 5:**
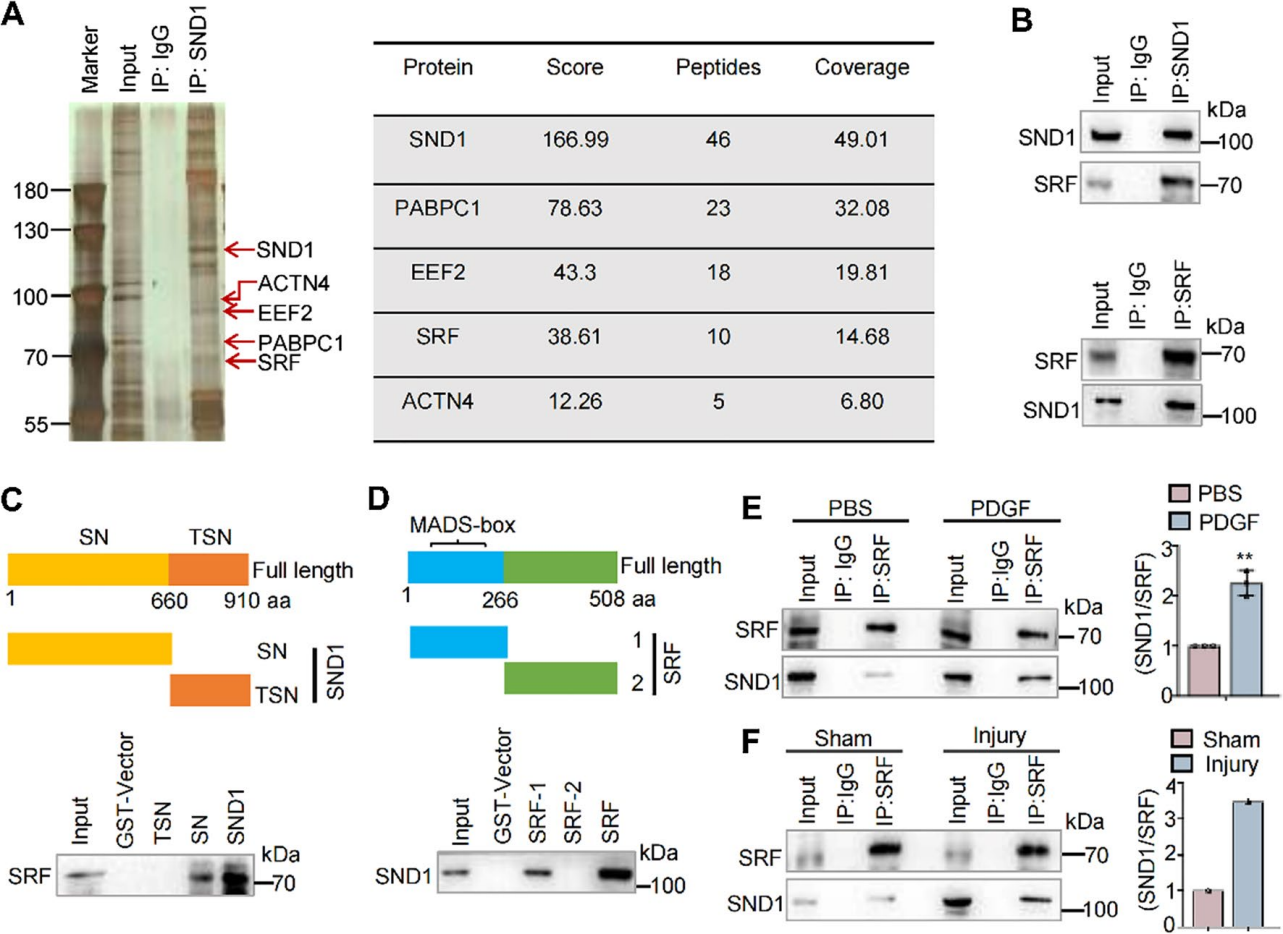
SND1 interacted with SRF. **A** Immuno-purification and mass spectrometry analysis of SND1-containing complexes. Cellular extracts (2 mg) from primary VSMCs were immunopurified using 30 µL protein A/G beads and the SND1 antibody. The eluants were resolved on SDS-PAGE and stained with silver. Mass spectrometry was used to examine the bands on the gel. **B** Primary VSMCs from mice were isolated and cultured, 1.5 × 10^7^ cells were harvested. Co-IP assay analysed the association between SND1 and SRF. **C** GST pull-down assay analysed the interaction of GST-fusion protein containing full-length SND1 (GST-SND1), SN domain (GST-SN), and TSN domain (GST-TSN) with the in vitro translated SRF. **D** GST pull-down assay analysed the interaction of GST-fusion protein containing full-length SRF (GST-SRF), SRF-1 domain (containing MADS box), and SRF-2 domain with His-SND1. **E** The primary VSMCs were cultured and seed into 10 cm dish, cells were allowed to grow until the cells were 70 to 80% confluent and treated with PDGF (40 ng/mL) for 24 h after being cultured in serum-free DMEM for 24 h, 1.5 × 10^7^ cells were collected for Co-IP assay with IgG or SRF antibody. **F** Wire-induced mouse femoral artery injury (*n* = 6 /group) models were generated. The femoral arteries were collected 28 days after the injury for Co-IP assay with IgG or SRF antibody. The immunoprecipitated complex was immunoblotted with SND1 and SRF antibodies. Western blotting results were analyzed by ImageJ (2 ×) software. Data are presented as the mean ± SD (*n* = 3). Statistical analysis was performed by unpaired two-tailed Student’s *t*-test. **P < 0.01

To determine the molecular mechanism by which the SND1 protein promotes the VSMC proliferative phenotype, we performed affinity purification and mass spectrometry to identify SND1-associated proteins from cellular extracts of primary VSMCs. Mass spectrometry revealed that SND1 was co-purified with several proteins, including the SRF protein (Fig. 5A). The SRF, a transcription factor, plays an important role in regulating differentiation and proliferation [7–9]. The physical interaction between SND1 and SRF was further verified by Co-IP assay in VSMCs. As shown in Fig. 5B, endogenous SND1 efficiently precipitated endogenous SRF and vice versa. We then used the GST pull-down assay to map the interaction domain between SND1 and SRF. The results showed that the full-length SND1 or SN domain was efficiently associated with SRF (Fig. 5C), while the full-length SRF or SRF-1 domain (containing the MADS-box) was efficiently associated with SND1 (Fig. 5D). Coomassie blue staining for GST-fusion proteins refers to Supplementary Fig. S5. To further clarify the binding of SRF and SND1 during VSMC phenotype switching, we examined the association between these two proteins with PDGF stimulation or under vascular injury. The results showed that the association between SND1 and SRF were enhanced with PDGF treatment in vitro (Fig. 5E) or after vascular injury in vivo (Fig. 5F).

The MYOCD is also a cofactor that binds with SRF to activate the expression of VSMC differentiation and contractile genes [22]. GST-pulldown assay revealed that MYOCD interacted with the MADS domain of SRF (Supplementary Fig. S6A) as previously reported [23]. As SND1 and MYOCD physically interact with the same binding domain of SRF, we speculated that SND1 and MYOCD competitively bind with SRF to regulate VSMC phenotype switching. The Co-IP assay demonstrated that the association between MYOCD and SRF was impaired by the overexpression of SND1 or PDGF treatment (Supplementary Fig. S6, B and C). Moreover, the mRNA levels of differentiation genes (*Acta2* and *Myh11*) were increased in the primary VSMCs from *Snd1-F/F Tagln-Cre* mice compared with *Snd1-F/F* mice, while decreased in the primary VSMCs that were infected with pLVX-IRES-SND1 compared with vector group (Supplementary Fig. S6D). These explained why the protein levels of differentiation genes increased upon SND1 depletion (Fig. 4A) and decreased upon SND1 overexpression (Supplementary Fig. S4A). These results indicated that SND1 physically interacts with SRF and hinders the binding of SRF-MYOCD.

**Fig. 6:**
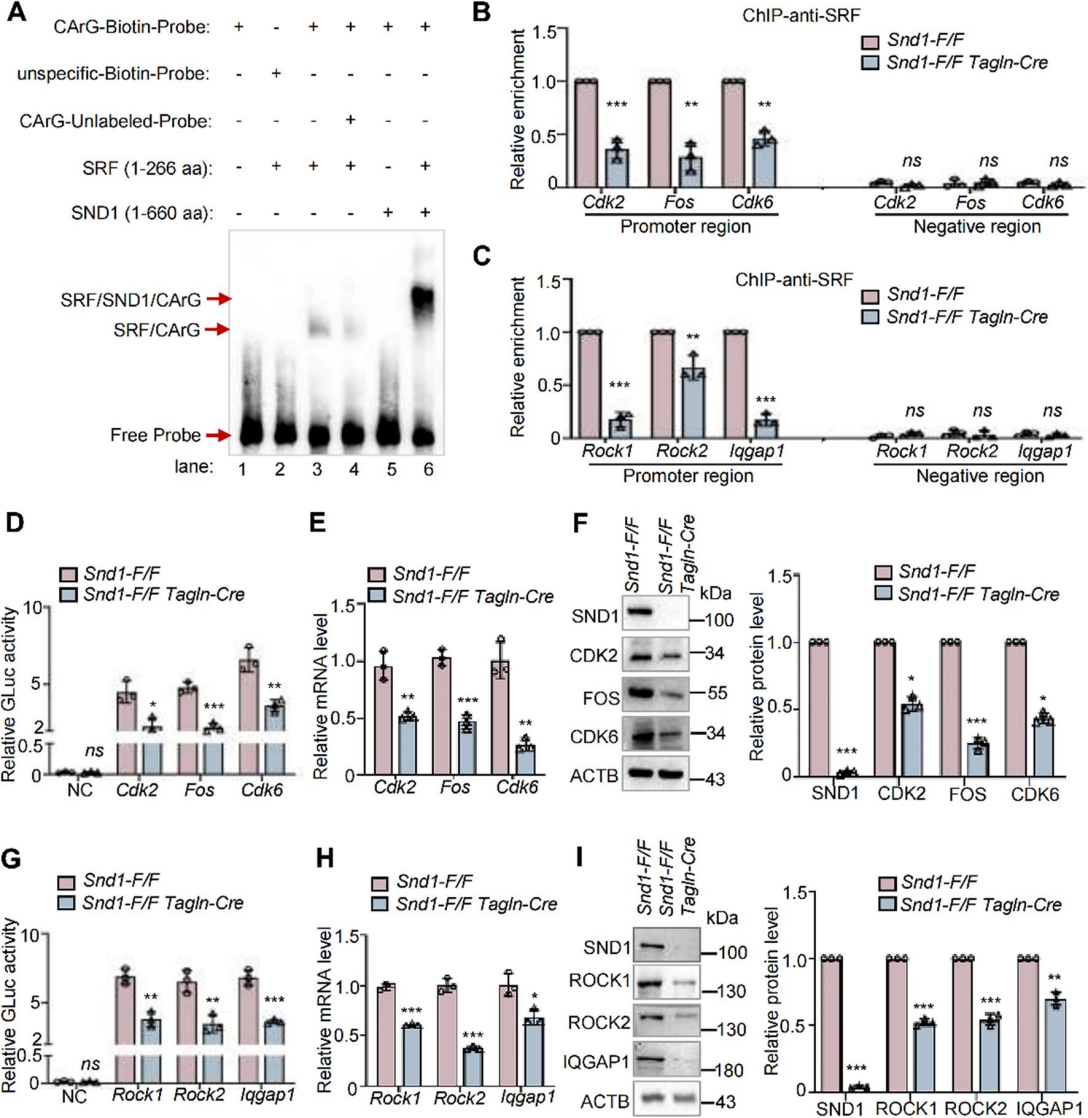
SND1 bound with SRF-CArG complex to promote VSMC proliferation- and migration-related gene transcription. **A** EMSA assays were performed using purified SRF1(1–266 aa, 0.1 μg), or SND1 (1–660 aa, 0.1 μg) protein and Biotin-labeled probes targeting the CArG motif (–CCATATTAGG–, 20 fmoL) of the *Fos* gene. Unlabeled- or unspecific probes were used for competition experiments. **B**, **C** 100 μg of cell nuclear lysate were collected from 1.5 × 10^7^ primary *Snd1-F/F* and *Snd1-F/F Tagln-Cre* VSMCs. SRF enrichments at *Cdk2*, *Fos*, *Cdk6*, *Rock1*, *Rock2*, and *Iqgap1* promoter were analyzed by ChIP assay. **D**, **G** The *Snd1-F/F* and *Snd1-F/F Tagln-Cre* VSMCs were allowed to grow until the cells were 70% confluent (2 × 10^5^ per well in 6-well plates) and infected with the GLuc-*Cdk2*, GLuc-*Fos*, GLuc-*Cdk6*, GLuc-*Rock1*, GLuc-*Rock2*, and GLuc-*Iqgap1* promoter plasmids, empty GLuc-Vector as the negative control (NC). Relative luciferase activity was evaluated by the ratio of GLuc / SEAP activity. 2 × 10^6^ primary VSMCs were obtained in a 6 cm dish. **E**, **H** The mRNA levels of *Cdk2*, *Fos*, *Cdk6, Rock1*, *Rock2*, and *Iqgap1* were detected by RT-qPCR. **F**, **I** The protein levels of SND1, CDK2, FOS, CDK6, ROCK1, ROCK2, IQGAP1 and ACTB were detected by western blotting. Western blotting results were analyzed by ImageJ (2 ×) software. Data are presented as the mean ± SD (*n* = 3). Statistical analysis was performed by unpaired two-tailed Student’s *t* test. *P < 0.05; **P < 0.01; ***P < 0.001; *ns* no significance

To verify whether SND1 acts as a co-activator of SRF, SRF-related proliferation genes (*Cdk2, Fos,* and *Cdk6*) and migration genes (*Rock1, Rock2,* and *Iqgap1*) were selected as the target genes according to the SRF ChIP-Seq data [9, 24]. It is well established that SRF binds with the CArG motif, a 10-bp element (consensus CC(A/T)^6^GG), allowing one base pair mismatch [25]. As shown in Supplementary Fig. S7A, all of these genes (*Fos, Cdk2, Cdk6, Rock1, Rock2,* and *Iqgap1*) contain the CArG motif. To test whether SND1 binds to the SRF-CArG complex, electrophoretic mobility shift assay (EMSA) assay was performed using purified SRF (1–266 aa) and SND1 (1–660 aa)*,* and the biotin-labeled/unlabeled probes targeting the classic CArG motif (–CCATATTAGG–) of the *Fos* gene. As shown in Fig. 6A, SRF bound to the CArG motif probes (lane 3), and the unlabeled probes diminished the binding of SRF with the labeled probes (lane 4). There was no binding of SND1 to the CArG motif probes (lane 5); however, SND1 and SRF formed a complex with the CArG motif (lane 6). It indicates that the interaction of SND1 and the CArG motif depends on the SRF and proves that SND1 and SRF form a complex with the CArG motif. Furthermore, we designed probes targeting other CArG motifs of proliferation and migration genes and obtained similar results (Supplementary Fig. S7, B, C, and D).

ChIP assays were performed to detect whether SND1 influences the binding ability of SRF to the promoter region, and the primers were designed to target the CArG motif containing promoter regions of these genes. The results showed that the knockout of SND1 reduced the binding of SRF to the promoter regions of proliferation (*Cdk2, Fos,* and *Cdk6*) (Fig. 6B) and migration (*Rock1, Rock2,* and *Iqgap1*) genes (Fig. 6C). In addition, the dual-reporter system was used to detect the transcriptional activity of the SRF target genes. The result showed that knockout of *Snd1* significantly reduced the transcriptional activity of SRF target proliferation genes (*Cdk2, Fos,* and *Cdk6*) (Fig. 6D) and migration genes (*Rock1, Rock2,* and *Iqgap1*) (Fig. 6G). Meanwhile, the mRNA and protein levels of these genes were decreased in primary *Snd1-F/F Tagln-Cre* VSMCs (Fig. 6E, F, H, and I).

**Fig. 7:**
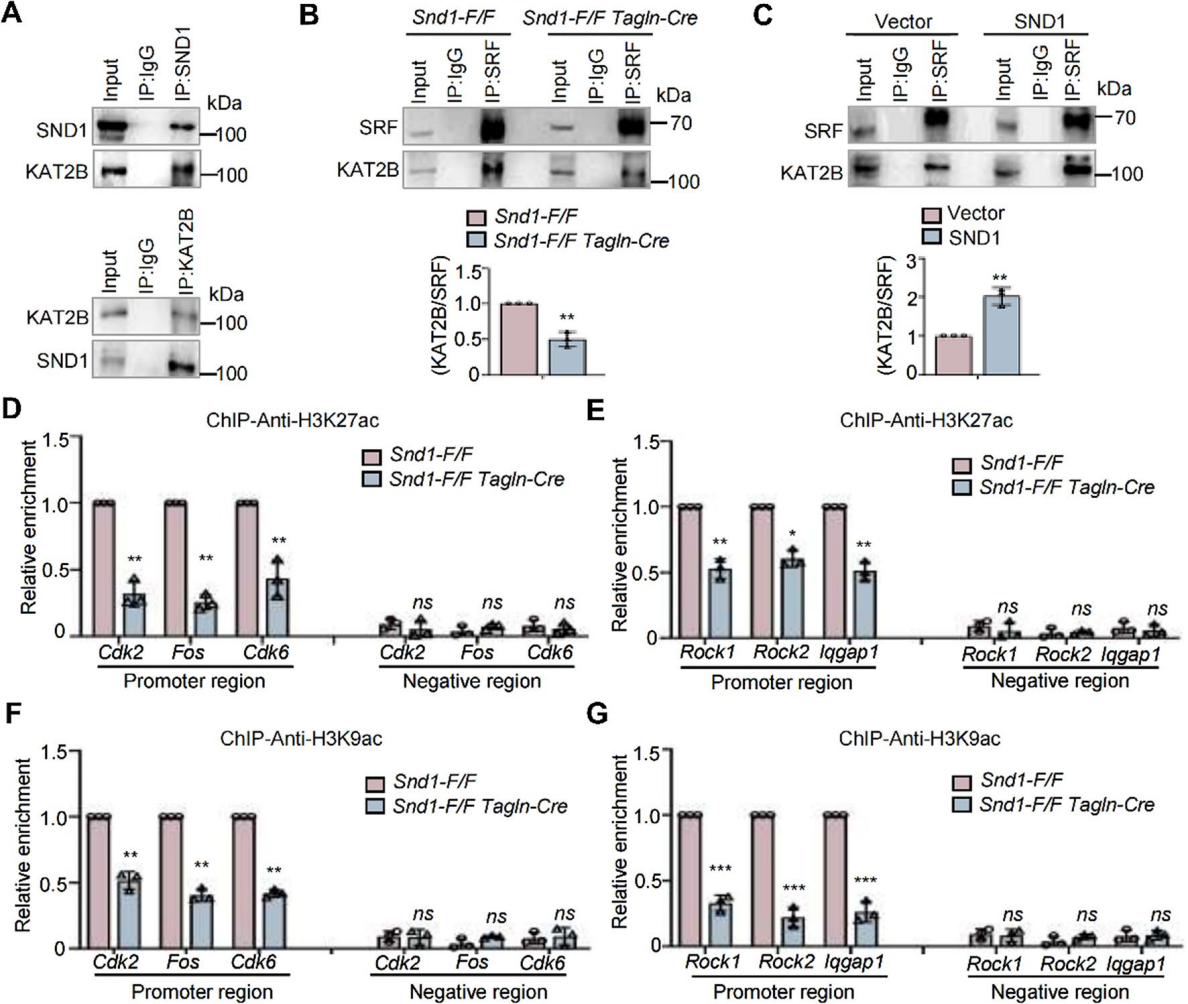
SND1 acted as a co-activator of SRF by recruiting KAT2B. **A** The primary VSMCs were cultured and seed into 10 cm dish, 1.5 × 10^7^ cells were collected. The association between SND1 and KAT2B was analyzed by Co-IP. **B** The association between SRF and KAT2B in primary *Snd1-F/F* and *Snd1-F/F Tagln-Cre* VSMCs were analyzed by Co-IP. **C** The primary VSMCs were allowed to grow until the cells were 70 to 80% confluent and infected with lentiviral pLVX-IRES-Vector or pLVX-IRES-FLAG-SND1, 1.5 × 10^7^ cells from each group were collected. Co-IP analyzed the association between SRF and KAT2B. All western blotting results were analyzed by ImageJ (2 ×) software. 100 μg of cell nuclear lysate were collected from primary *Snd1-F/F* and *Snd1-F/F Tagln-Cre* VSMCs. H3K27ac (**D**) and H3K9ac (**F**) enrichment at *Cdk2*, *Fos*, and *Cdk6* promoter in *Snd1-F/F* and *Snd1-F/F Tagln-Cre* VSMCs were analyzed by ChIP. H3K27ac (**E**) and H3K9ac (**G**) enrichment at *Rock1*, *Rock2*, and *Iqgap1* promoter in *Snd1-F/F* and *Snd1-F/F Tagln-Cre* VSMCs were analyzed by ChIP. Data are presented as the mean ± SD (*n* = 3). Statistical analysis was performed by unpaired two-tailed Student’s t-test. *P < 0.05; **P < 0.01; ***P < 0.001; *ns* no significance

In our previous study, we showed that SND1 acted as a co-activator by recruiting histone lysine acetyltransferases (HATs), such as lysine acetyltransferase 2B (KAT2B) (also known as GCN5), to transcription complexes which facilitating histone acetylation and chromatin accessibility [11]. Therefore, we examined the physical association of SND1 and KAT2B in VSMCs by Co-IP assay. As shown in Fig. 7A, endogenous SND1 interacted with KAT2B and vice versa. We also detected whether SND1 influenced the interaction between SRF and KAT2B. The results showed that knockout of *Snd1* in *Snd1-F/F Tagln-Cre* VSMCs diminished the interaction between SRF and KAT2B (Fig. 7B), while overexpression of SND1 increased SRF-KAT2B interaction (Fig. 7C). Moreover, acetyl-histone ChIP assay revealed that the enrichment of acetyl-histone H3 Lys27 (H3K27ac) and acetyl-histone H3 Lys9 (H3K9ac) were considerably reduced at the proliferation (*Cdk2, Fos,* and *Cdk6*) and migration (*Rock1, Rock2,* and *Iqgap1*) gene promoters with SND1 loss-of-function (Fig. 7D, E, F, and G). These data indicate that SND1 acts as a co-activator that regulates SRF-mediated proliferation- and migration-related gene transcriptional activation by recruiting KAT2B.

### ELK1 promotes transcriptional activation of *Snd1* in VSMC proliferation

**Fig. 8:**
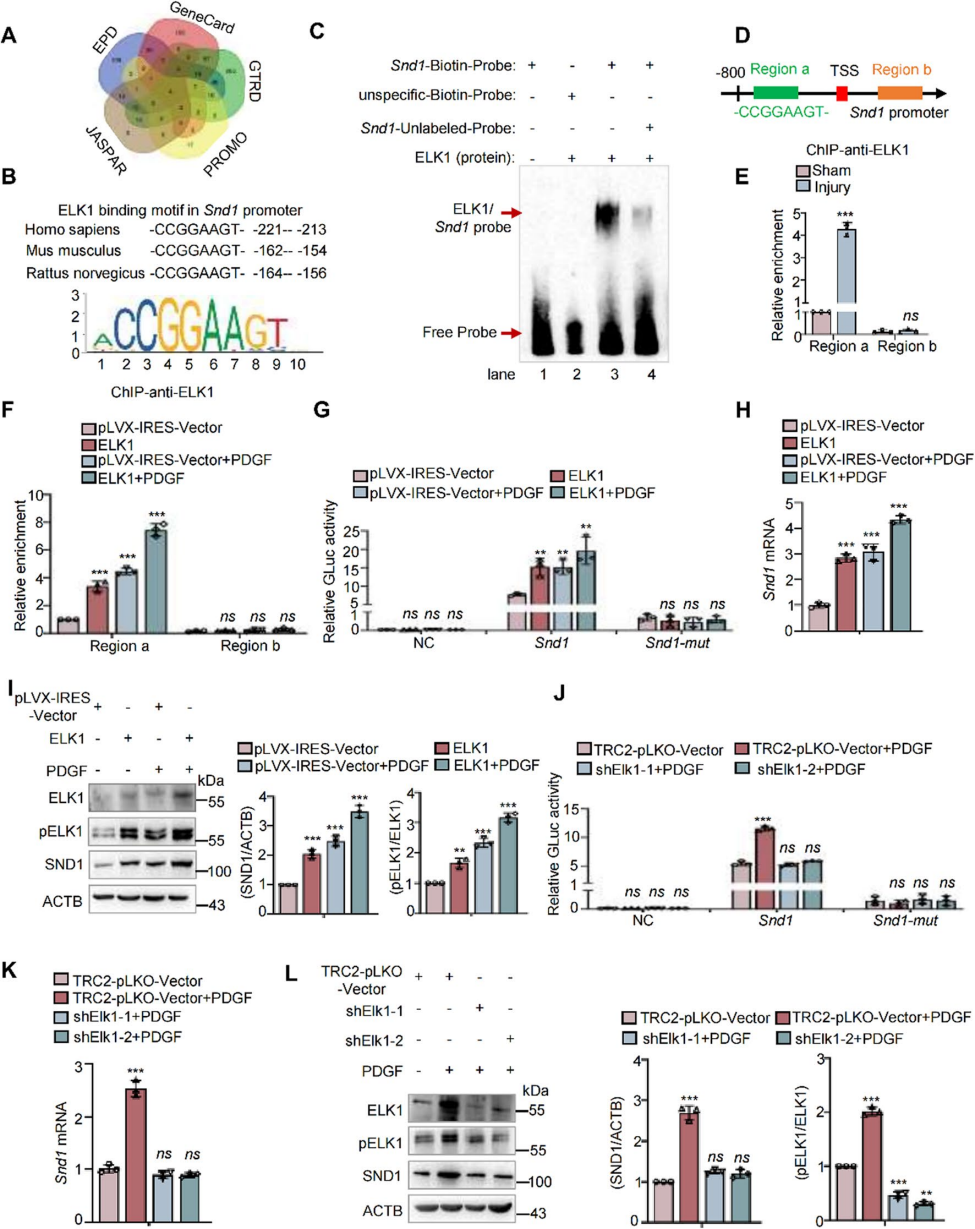
ELK1 promoted transcriptional activation of *Snd1* in VSMC proliferation. **A** Venn diagram showed the upstream transcription factors of *Snd1* predicted by JASPAR, EPD, GeneCard, GTRD, and PROMO bioinformatics software. **B** ELK1 binding motif (–CCGGAAGT–) in *Snd1* promoter region across vertebrates was analyzed by the JASPAR database.** C** EMSA assays were performed using purified ELK1 (0.1 μg) protein and Biotin-labeled probes targeting motif (–CCGGAAGT–, 20 fmoL) of the *Snd1*, unlabeled- or unspecific probes were used for competition experiments. **D** Primers were designed for ChIP based on the promoter regions containing the ELK1 binding motif (Region a), the region located outside the promoter region (Region b) as the negative control. **E** The mice femoral arteries were obtained after the injury (*n* = 8 /group), ChIP analysis of ELK1 enrichment at different regions of *Snd1* promoter. **F** 100 μg of cell nuclear lysate were collected by 1.5 × 10^7^ primary cells for ChIP assay. ChIP analysis of ELK1 enrichment at different regions of *Snd1* promoter in VSMCs that infected with pLVX-IRES-ELK1 or pLVX-IRES-Vector and stimulated with or without PDGF (40 ng/mL) for 24 h. **G**,** J** The primary VSMCs were allowed to grow until the cells were 70% confluent (2 × 10^5^ per well in 6-well plates). Then primary VSMCs were infected with the GLuc plasmids carrying wild-type *Snd1* promoter (− 2500 to + 500 bp) or *Snd1* promoter with ELK1 binding site mutant (*Snd1*-mut), empty GLuc-Vector as the negative control (NC), together with pLVX-IRES-ELK1, or pLVX-IRES-Vector plasmids, or TRC2-pLKO-shElk1-1, TRC2-pLKO-shElk1-2, or TRC2-pLKO-Vector, with or without PDGF treatment for 24 h. Relative luciferase activity was evaluated by the ratio of GLuc / SEAP activity. **H**, **I** The primary VSMCs were infected with pLVX-IRES-ELK1 or pLVX-IRES-Vector and then were stimulated with or without PDGF (40 ng/mL) for 24 h, 2 × 10^6^ primary VSMCs were obtained in a 6 cm dish. The mRNA levels of *Snd1* were detected by RT-qPCR. The protein levels of SND1, ELK1, pELK1 and ACTB in were detected by western blotting. **K**–**L** The primary VSMCs were infected with TRC2-pLKO-Vector, shElk1-1 or shElk1-2 and then stimulated with or without PDGF (40 ng/mL) for 24 h, 2 × 10^6^ primary VSMCs were obtained in a 6 cm dish. The mRNA levels of *Snd1* were detected by RT-qPCR. The protein levels of SND1, ELK1, pELK1 and ACTB were detected by western blotting. Data are presented as the mean ± SD (*n* = 3). Statistical analysis was performed by one-way ANOVA (from E, F, H, I, K, and L), or three-way ANOVA (G, J) followed by Bonferroni post-hoc correction. **P < 0.01; ***P < 0.001; *ns* no significance

To delineate the underlying mechanism of SND1 upregulation in proliferating VSMCs, we used bioinformatics analyses, such as the JASPAR, EPD, GeneCard, GTRD, and PROMO databases, to predict the putative transcription factors that recognize the promoter region of *Snd1* (Fig. 8A). ELK1 is one of the predicted transcription factors. In addition, JASPAR database analysis indicated that the *Snd1* promoter region contains ELK1 binding motif (–CCGGAAGT–) which is conserved across vertebrates (Fig. 8B). ELK1 is a member of the ternary complex factor (TCF) subfamily of ETS transcription factors, and is directly activated through phosphorylation in response to activating the PDGF/ERK signaling pathway, which plays essential roles in regulating gene expression during VSMC phenotype switching [26]. To test whether ELK1 binds to the specific motif of *Snd1* promoter and promotes the transcription activity of *Snd1* in this process, EMSA assay was performed using purified ELK1 protein and the biotin-labeled/unlabeled probes targeting the motif (–CCGGAAGT–) of the *Snd1*. As shown in Fig. 8C, ELK1 bound to the specific motif probes (lane 3), and the unlabeled probes diminished the binding of ELK1 with the labeled probes (lane 4). Furthermore, ChIP analysis was performed to detect the enrichment of ELK1 in *Snd1* promoter under vascular injury or with PDGF stimulation. Primers were designed for ChIP based on the promoter regions containing the ELK1 binding motif (Region a). The region located outside the promoter region (Region b) was used as a negative control (Fig. 8D). As expected, ELK1 proteins were enriched in the *Snd1* promoter regions and the enrichment was increased under vascular injury (Fig. 8E) or with PDGF stimulation (Fig. 8F). The dual-luciferase reporter assay was performed to investigate whether ELK1 regulates the transcriptional activity of *Snd1*. The result showed that the activity of GLuc-*Snd1* promoter was enhanced by PDGF treatment or ELK1 overexpression, and the enhancement was more significant with PDGF and ELK1 together (Fig. 8G). Moreover, the mRNA and protein levels of SND1 were detected when we increased the ELK1 expression or activity. The result showed that overexpression of ELK1 or PDGF stimulation upregulated the mRNA and protein levels of SND1, and the upregulation was more significant with PDGF stimulation and ELK1 overexpression together (Fig. 8H and I). In contrast, the PDGF-induced increased transcriptional activity of *Snd1* was inhibited by the knockdown of ELK1 with shRNA (Fig. 8J), as well as the mRNA and protein levels of SND1 (Fig. 8K and L). These findings support the hypothesis that ELK1 is a novel transcription factor that regulates the expression of SND1 during PDGF stimulation.

### Inhibition of the phosphorylation of ELK1 by U0126 retards injury-induced SND1 upregulation and neointima hyperplasia

In this study, we noticed that both the phosphorylated and the whole protein levels of ELK1 were increased after PDGF simulation (Fig. 8I and L). We further detected the expression and phosphorylation level of ELK1 during VSMCs phenotypic switching using vascular injury models. The results showed that ELK1 was highly expressed in neointima comparing with normal rat carotid or mice femoral artery, as well as the pELK1 (Supplementary Fig. S8, A and C). Western blotting also showed that the whole protein level and phosphorylated ELK1 are both increased in the injured arteries comparing with the sham-operated control (Supplementary Fig. S8, B and D). Consistently, the cultured aorta presented the same result (Supplementary Fig.S8E). These results suggested that the upregulated ELK1/pELK1 during vascular injury results in the upregulation of SND1.

**Fig. 9:**
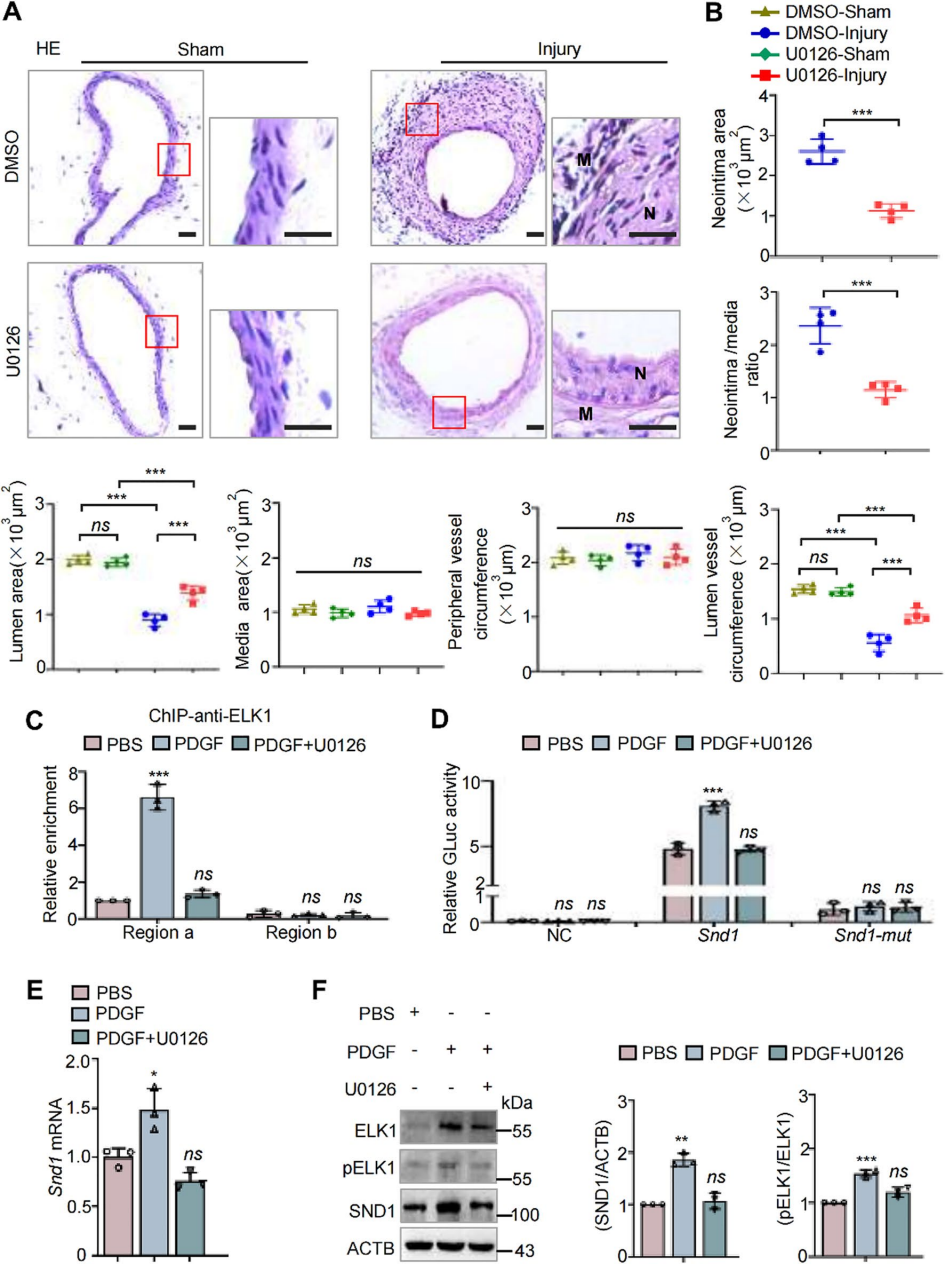
Inhibition of the phosphorylation of ELK1 by U0126 retards injury-induced SND1 upregulation and neointima hyperplasia. **A** Wire-induced mouse femoral artery injury models were constructed, and U0126 was injected intraperitoneally every 3 days (1 mg/kg) after wire injury. The femoral arteries were collected (*n* = 4 /group). Representative HE staining of sham-operated and wire-injured femoral arteries from DMSO and U0126 treated mice. (*N* neointima; *M* media) **B** Averaged data of the neointima area, neointima/media ratio, lumen area, media area, peripheral vessel circumference, lumen vessel circumference of sham-operated or wire-injured femoral arteries (*n* = 4 /group). **C** 100 μg of cell nuclear lysate were collected by 1.5 × 10^7^ primary cells for ChIP assay. ChIP analysis of ELK1 enrichment at different regions of *Snd1* promoter in primary VSMCs with PDGF in the presence or absence of U0126 (10 mM). **D** The primary VSMCs were allowed to grow until the cells were 70% confluent (2 × 10^5^ per well in 6-well plates). Then cells were infected with the GLuc plasmids carrying wild-type *Snd1* promoter (− 2500 to + 500 bp) or *Snd1* promoter with ELK1 binding site mutant (*Snd1*-mut), empty GLuc-Vector as the negative control (NC), together with PDGF in the presence or absence of U0126 (10 mM). Relative luciferase activity was evaluated by the ratio of GLuc/SEAP activity. **E**, **F** The primary VSMCs were allowed to grow until the cells were 70% confluent and were stimulated with or without PDGF for 24 h in the presence or absence of U0126 (10 mM) for 24 h. The mRNA levels of *Snd1* were detected by RT-qPCR. The protein levels of SND1, ELK1, pELK1 and ACTB were detected by western blotting. All western blotting results were analyzed by ImageJ (2 ×) software. Scale bar, 100 μm. Data are presented as the mean ± SD (*n* = 3). Statistical analysis was performed by unpaired two-tailed Student’s *t*-test (A), one-way ANOVA (C, E, and F) or three-way ANOVA (A and D) followed by Bonferroni post-hoc correction. *P < 0.05; **P < 0.01; ***P < 0.001; *ns* no significance

U0126 has been used as an efficient inhibitor of the ERK/ELK1 signaling pathway [26]. To further verify the function of the ELK1/SND1 pathway during the progression of neointimal hyperplasia, we performed the wire-induced mouse femoral artery injury model with or without the U0126 treatment. The results showed that the phosphorylation of ELK1 and the upregulation of SND1 in the injured femoral arteries were inhibited by U0126 treatment (Supplementary Fig. S9), meanwhile, the injury-induced pronounced neointimal hyperplasia was inhibited by the U0126 treatment (Fig. 9A and B). Furthermore, we treated the primary VSMCs with PDGF in the presence or absence of U0126. The result showed that treatment with U0126 interrupted the efficient binding of ELK1 to the *Snd1* promoter region (Fig. 9C) and reduced transcriptional activation of *Snd1* (Fig. 9D). Moreover, U0126 inhibited the phosphorylation of ELK1 and prevented the enhancement of SND1 expression induced by PDGF (Fig. 9E and F). These findings further suggest that U0126-induced SND1 downregulation is a potential therapeutic strategy to suppress the neointimal hyperplasia and vascular stenosis.

## Discussion

Phenotype switching of VSMCs from a differentiated contractile to a dedifferentiated proliferative phenotype is a key step in the formation of neointima, a hallmark of several prominent vascular diseases [27]. Consistent with this notion, we also found that most neointimal cells derived from mature VSMCs displayed decreased VSMC contractile proteins compared to medial layer contractile VSMCs. Interestingly, we found that these phenotype-switched proliferative VSMCs exhibited higher expression of SND1 protein, together with similar ex vivo findings in cultured VSMCs. This suggests that SND1 is a novel regulator associated with the proliferative VSMC phenotype, predisposing vessels to pathological remodeling, such as neointimal hyperplasia.

SND1 is a conserved and multifunctional protein that is highly expressed in proliferating cells but not in differentiated cells [11, 12]. We found that vascular injury in mice significantly increased SND1 expression in VSMCs, which was closely related to VSMC phenotype switching. The increased PCNA and decreased ACTA2, CNN1, and MYH11 levels in the injured arteries supported the switch of VSMCs from a contractile state to a proliferative phenotype. PDGF, which is secreted by damaged endothelium cells and immune cells during vascular injury, is a powerful inducer of VSMC phenotype switching [20]. In this study, we found that PDGF stimulation enhances SND1 expression in VSMCs. To further clarify the SND1 expression change in human vascular diseases, we analyzed the RNA-seq data from the human atherosclerotic carotid artery. The results showed that SND1 was upregulated in the plaque rupture areas compared with normal adjacent areas [28]. Moreover, SND1 was downregulated during vascular physiological development when VSMCs gradually differentiated from the initial proliferative and synthetic phenotype to a differentiated and contractile state (Supplementary Fig. S2). These findings demonstrate a strong association between the expression level of SND1 and the VSMC phenotype, thereby implying the crucial involvement of SND1 in vascular disease.

The phenotype switching of VSMC is controlled by a series of transcription factors and their co-factors. SRF is a ubiquitously expressed transcription factor that plays crucial role in regulating proliferative and contractile VSMC phenotypes via its association with different co-factors [9]. As reported, SRF acts in partnership with MYOCD to activate VSMC differentiation marker gene transcription, whereas ELK1 enhances the transcription of the VSMC proliferation marker gene transcription [29]. Phosphatase and tensin homolog (PTEN), a multifunctional tumor suppressor, also functions as an essential co-factor of SRF that activates SRF-dependent VSMC differentiation gene transcription [30]. The basic helix-loop-helix transcription factor (TCF21) interacts with SRF and inhibits VSMC differentiation by disrupting the formation of the SRF/MYOCD complex [24]. In this study, SND1 was identified as a co-activator of SRF that did not interact with the promoter region of the proliferation- and migration-genes directly, but function through binding with SRF. As a co-activator of SRF, SND1 facilitates histone acetylation and chromatin accessibility in the promoter region of proliferation- and migration-related genes by recruiting the histone acetyltransferase, KAT2B, allowing SRF to recognize the CArG motif of these genes and activates their transcription. Moreover, the interaction between SND1 and SRF hinders the binding between SRF and MYOCD, and subsequently inhibits the differentiation genes expression and reduced the contraction ability of VSMCs. These indicate that SND1 not only promotes VSMC proliferation and migration but also inhibits the contraction ability of VSMC, thus plays an essential role in the VSMC phenotypic switching. How to regulate different co-factors to competitively associate with SRF to activate different gene transcription is largely unknown, we are still investigating the underlying molecular mechanisms.

We then investigated the underlying molecular mechanism by which SND1 regulates neointimal hyperplasia. We predicted putative transcription factors of SND1 using bioinformatics analysis and found that ELK1 could enhance *Snd1* transcription activity in VSMCs. Although it has been reported that SP1 and NFYA are transcription factors that regulate the transcription activity of *Snd1* in cancer cells [31], in this study, we demonstrated that both of them did not affect *Snd1* transcription activity in VSMCs (Supplementary Fig. S10). ELK1 is a member of the ETS transcription factor family and is directly activated through phosphorylation in response to activating the PDGF/ERK signaling pathway [26], and plays a pivotal role in regulating the transcription of genes involved in VSMC differentiation or proliferation [9]. Here, we demonstrate that the stimulation of PDGF or vascular injury upregulates the expression of ELK1 and activates ELK1 through phosphorylation, then ELK1 acts as a novel transcription factor via binding with the ELK1-binding motif (-CCGGAAGT-) embedded in the *Snd1* promoter region. As a result, ELK1 promotes the transcriptional activation of *Snd1.* Consequently, the upregulated SND1 accelerates VSMC proliferation by targeting proliferative and migratory gene transcription by acting as a co-activator of the key transcription factor SRF in VSMCs. Although ELK1 is thought to mainly function through cooperation with the SRF, there are several instances that ELK1 binding occurs in the absence of SRF binding to the same promoter [32, 33]. In this study, we noticed that ELK1 regulated *Snd1* transcription without collaborating with SRF (Supplementary Fig. S11)*,* whereas ELK1 induced high expression of SND1 to associate with SRF to activate the transcriptional activation of proliferation and migration genes. These results reveal a new mechanism of ELK1 in cooperation with SRF to regulate gene transcription.

Although SND1 has been reported as a double-edged regulator under various contexts [34–36], this study demonstrates the pathological role of SND1 in injury-induced neointimal hyperplasia. We provide direct evidence for the functional role of SND1 in VSMCs in vivo by generating the SMC-specific *Snd1-F/F Tagln-Cre* mouse model, which indicates that *Snd1* knockout dramatically inhibits neointimal hyperplasia in a vascular injury model, suggesting that SND1 mediated VSMC proliferation and migration play a critical role in intimal thickening. Inhibition of proliferation-related signaling pathways is an important strategy to prevent VSMC phenotype switching and neointimal hyperplasia. Rapamycin, a classical inhibitor of mammalian target of rapamycin (mTOR), and its analogs (everolimus, biolimusA9, and zotarolimus) have been exploited in drug-eluting stents to inhibit the proliferation and migration of VSMCs and to make a breakthrough in the prevention of restenosis [37]. In this study, we identified that U0126, the ERK/ELK1 inhibitor, could prevent SND1 expression through inhibiting the ELK1 phosphorylation, and consequently inhibited the injury-induced pronounced neointimal hyperplasia. These findings suggest that U0126-induced SND1 downregulation is a potential therapeutic strategy to suppress neointimal hyperplasia and restenosis.

In summary, this study provides novel insights into the underlying mechanisms of VSMC phenotype switching and neointimal hyperplasia. We demonstrated that ELK1/SND1/SRF is a novel signaling pathway involved in VSMC phenotype switching and is a potential target for preventing neointimal hyperplasia and vascular stenosis.

### Supplementary Information

Below is the link to the electronic supplementary material.

Supplementary file1 (DOCX 8638 KB)

## Data Availability

The data for this study are available by contacting the corresponding author upon reasonable request.

## Abbreviations

ACTA2: α-Smooth muscle actin
ACTB: Beta-actin
Co-IP: Co-immunoprecipitation
CDK2: Cyclin-dependent kinase 2
CDK6: Cyclin-dependent kinase 6
ChIP: Chromatin immunoprecipitation assay
CCK-8: Cell counting kit-8
CNN1: Calponin
DMEM: Dulbecco’s modified Eagle’s medium
EdU: 5-Ethynyl-2′-deoxyuridine
ELK1: ETS transcription factor ELK1
EMSA: Electrophoretic mobility shift assay
ERK: Extracellular signal-regulated kinase
ETS: E26 transformation-specific
FBS: Fetal bovine serum
FOS: Fos proto-oncogene
HE: Hematoxylin-eosin
IQGAP1: IQ motif containing GTPase activating protein 1
MYH11: Smooth muscle myosin heavy chain
MYOCD: Myocardin
PBS: Phosphate buffer saline
PDGF: Platelet-derived growth factor
PCNA: Proliferating cell nuclear antigen
PCR: Polymerase chain reaction
RT-qPCR: Real-time quantitative polymerase chain reaction
ROCK1: Rho-associated coiled-coil containing protein kinase 1
ROCK2: Rho-associated coiled-coil containing protein kinase 2
shRNA: Short hairpin RNA
SRF: Serum response factor
SND1: Staphylococcal nuclease domain-containing protein 1
SD: Sprague-Dawley
TCF: Ternary complex factor
VSMC: Vascular smooth muscle cell
