# Supplementary material for: Vascular injury activates the ELK1/SND1/SRF pathway to promote vascular smooth muscle cell proliferative phenotype and neointimal hyperplasia
Source: Cell Mol Life Sci. 2024 Jan 27;81(1):59. doi: 10.1007/s00018-023-05095-x (PMC10817852; doi:10.1007/s00018-023-05095-x)
Supplement: Supplementary file 1 — Supplementary file1 (DOCX 8638 KB) [file 18_2023_5095_MOESM1_ESM.docx]

**Vascular injury activates the ELK1/SND1/SRF pathway to promote vascular smooth muscle cell proliferative phenotype and neointimal hyperplasia**

Chao Su^1, 3, 4†^, Mingxia Liu^2, 3, 4†^, Xuyang Yao^3, 4, 6†^, Wei Hao^2, 3, 4^, Jinzheng Ma^2, 3, 4^, Yuanyuan Ren^2, 3, 4^, Xingjie Gao^2, 3, 4^, Lingbiao Xin^2, 3, 4^, Lin Ge^2, 3, 4^, Ying Yu^3, 4^,

Minxin Wei^1^*, and Jie Yang^2, 3, 4, 5^*

^1^ Division of Cardiovascular Surgery, Cardiac and Vascular Center, The University of Hong Kong-Shenzhen Hospital, Shenzhen, China.

^2^ Department of Biochemistry and Molecular Biology, Department of Immunology, School of Basic Medical Science, Tianjin Medical University, Tianjin, China.

^3^ Key Laboratory of Immune Microenvironment and Disease (Ministry of Education), and Key Laboratory of Cellular and Molecular Immunology, Tianjin, China.

^4^ The Province and Ministry Co-sponsored Collaborative Innovation Center for Medical Epigenetics, Tianjin Medical University, Tianjin, China.

^5^ State Key Laboratory of Experimental Hematology, Tianjin, China.

^6^ Eye Institute & School of Optometry and Ophthalmology, Tianjin Medical University Eye Hospital, Tianjin, China.

^†^These authors contributed equally to this work.

*Correspondence: Minxin Wei, weimx@hku-szh.org. Jie Yang, yangj@tmu.edu.cn.

**Supplemental Figures**


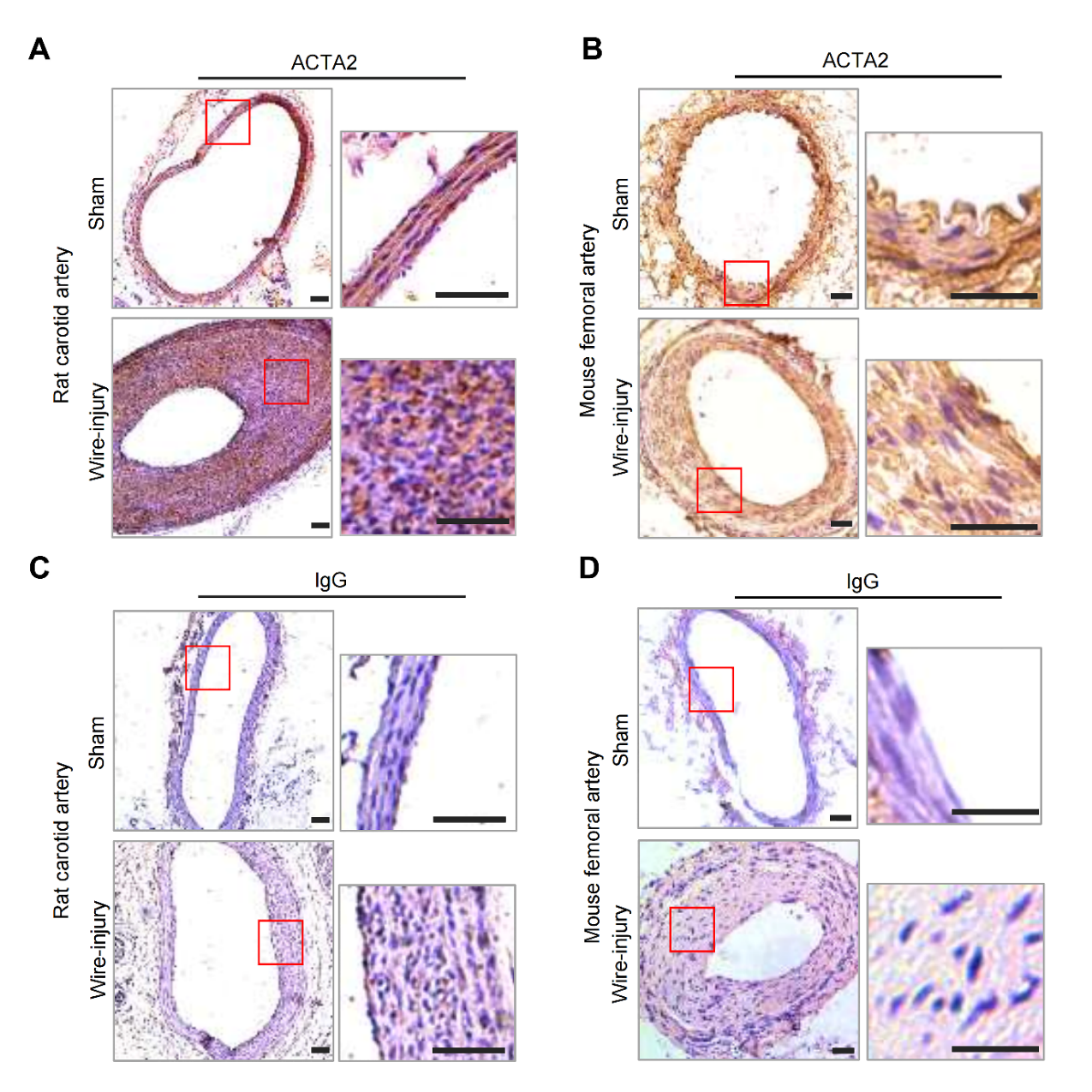


**Fig. S1 Positive and negative control of immunohistochemical staining in carotid and femoral arteries.** Wire-induced rat carotid artery injury (n = 6 /group) and mouse femoral artery injury (n = 6 /group) models were generated. The carotid and femoral arteries were collected at 14 or 28 days after the injury, respectively. **(A, B)** Immunohistochemical staining of ACTA2 in carotid or femoral arteries from sham-operated and wire-injured groups served as the positive control. **(C, D)** Immunohistochemical staining of rabbit IgG in carotid or femoral arteries from sham-operated and wire-injured groups served as the negative control. Scale bar, 200 μm in A and C; 100 μm in B and D.

**
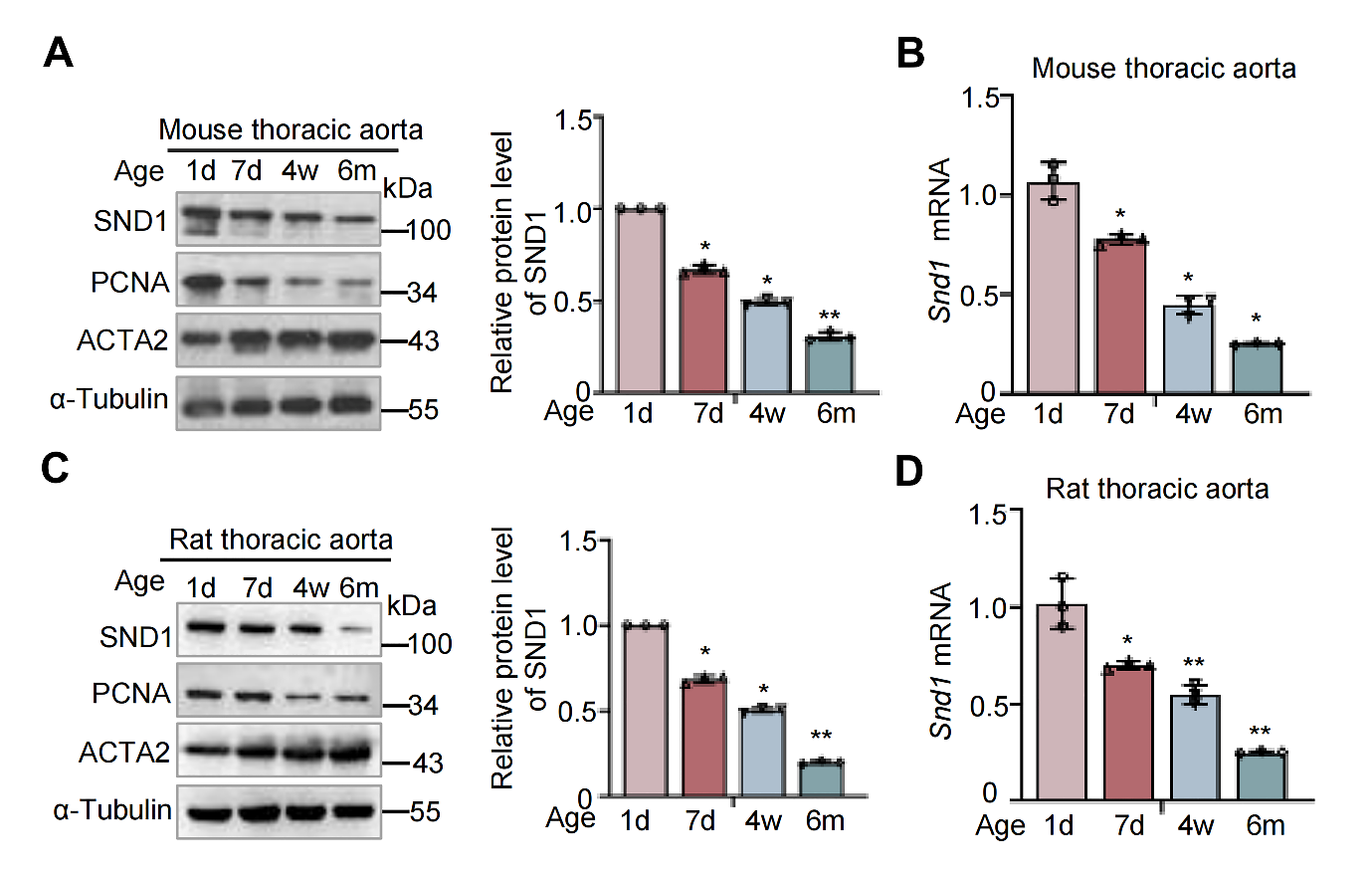
**

**Fig. S2 The expression pattern of SND1 in different phenotype of VSMCs.** Thoracic aortas were isolated from postnatal 1 day (1d), 7 days (7d), 4 weeks (4w), and 6 months (6m) mice or rats. **(A, C)** The protein levels of SND1, PCNA, ACTA2, and α-Tubulin were detected by western blotting, and **(B, D)** the mRNA levels of *Snd1* were detected by RT-qPCR. All western blotting results were analyzed by ImageJ (2×) software. Data are presented as the mean ± SD (n = 3). Results were analyzed by one-way ANOVA. **P* < 0.05; ***P* < 0.01.


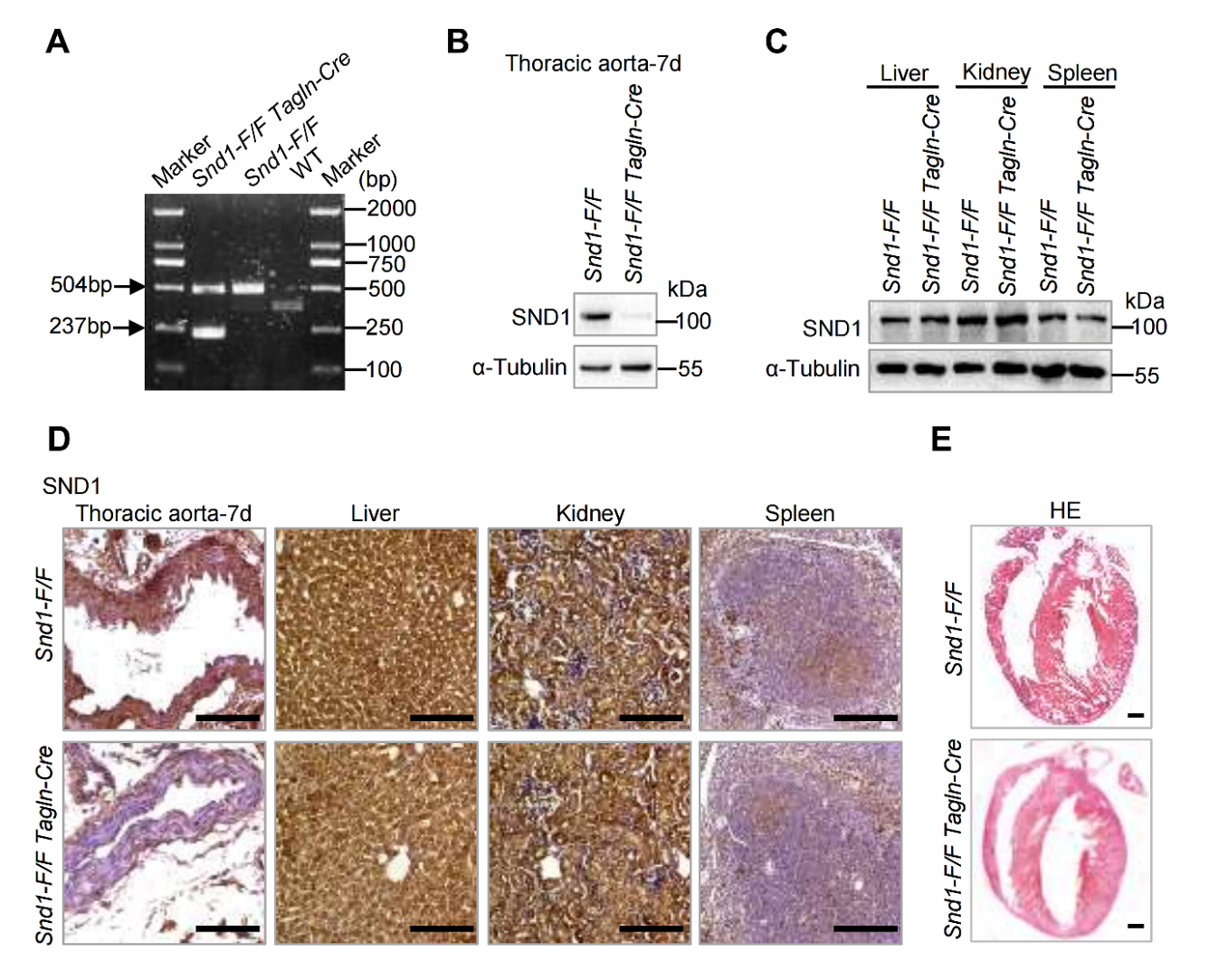


**Fig. S3 Construction and** [**verification**](javascript:;) **of *Snd1-F/F Tagln-Cre* mice. (A)** Genotyping of *Snd1-F/F* *Tagln-Cre*, *Snd1-F/F*, and Wild Type (WT) mice. A band of 504 bp indicates the product from *Snd1-F/F*, band of 237 bp indicates the product from *Snd1-F/F Tagln-Cre*. **(B)** SND1 expression levels in the thoracic aorta from 7-days-old (7d) *Snd1-F/F* and *Snd1-F/F* *Tagln-Cre* mice were detected by western blotting. **(C)** SND1 expression levels in liver, kidney, and spleen tissues of *Snd1-F/F* and *Snd1-F/F Tagln-Cre* mice were detected by western blotting. **(D)** Immunohistochemical staining of SND1 in thoracic aorta-7 d, liver, kidney, and spleen tissues of *Snd1-F/F* and *Snd1-F/F Tagln-Cre* mice. **(E)** Representative HE staining of heart from *Snd1-F/F* and *Snd1-F/F Tagln-Cre* mice Scale bar, 100 μm.

**
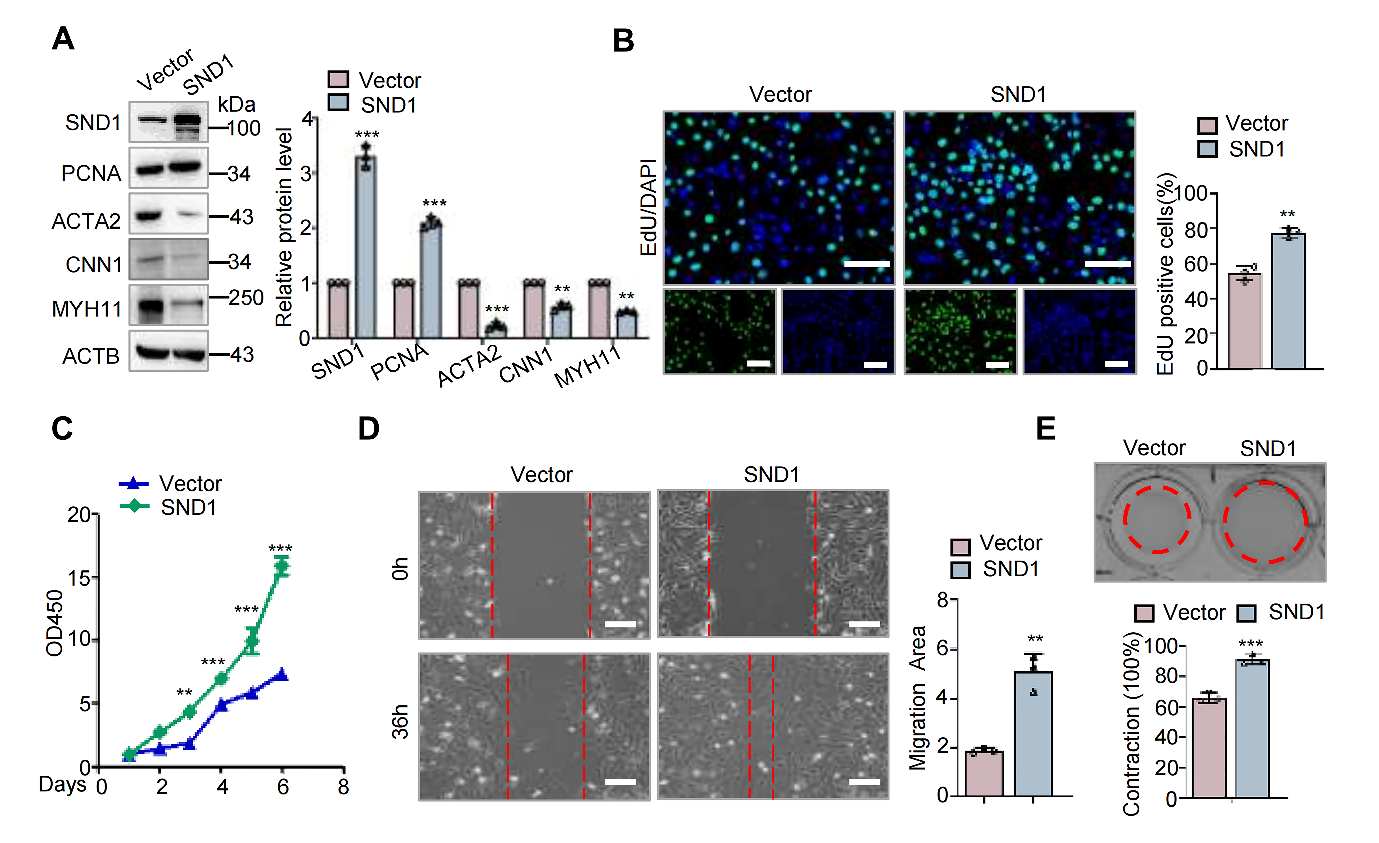
**

**Fig. S4 SND1 promoted VSMC proliferative phenotype.** The primary VSMCs were allowed to grow until the cells were 70 % confluent (2×10^5^ per well in 6-well plates) and then were infected with pLVX-IRES-SND1 or pLVX-IRES-Vector. **(A)** The protein levels of SND1, PCNA, ACTA2, CNN1, MYH11, and ACTB were detected by western blotting. All western blotting results were analyzed by ImageJ (2×) software. Cell proliferation ability was detected by **(B)** EdU incorporation (1×10^4^ cells in per 48 well plates) and **(C)** CCK-8 assay (5×10^3^ cells in per 96-well plates). **(D)** Cell migration ability was detected by wound-healing assays (3×10^5^ cells in per 6-well plates). **(E)** Cell contraction ability was detected by collagen gel contraction assay (2×10^4^ cells in per 12-well plates). % Contraction=Gel Area (cm^2^) /Well Area (cm^2^) ×100%. Scale bar, 100 μm. Data are presented as the mean ± SD (n = 3). Results in A, B, D, and E were analyzed by unpaired two-tailed Student’s t-test, results in C were analyzed by repeated measures ANOVA. **P* < 0.05; ***P* < 0.01; ****P* <0.001.


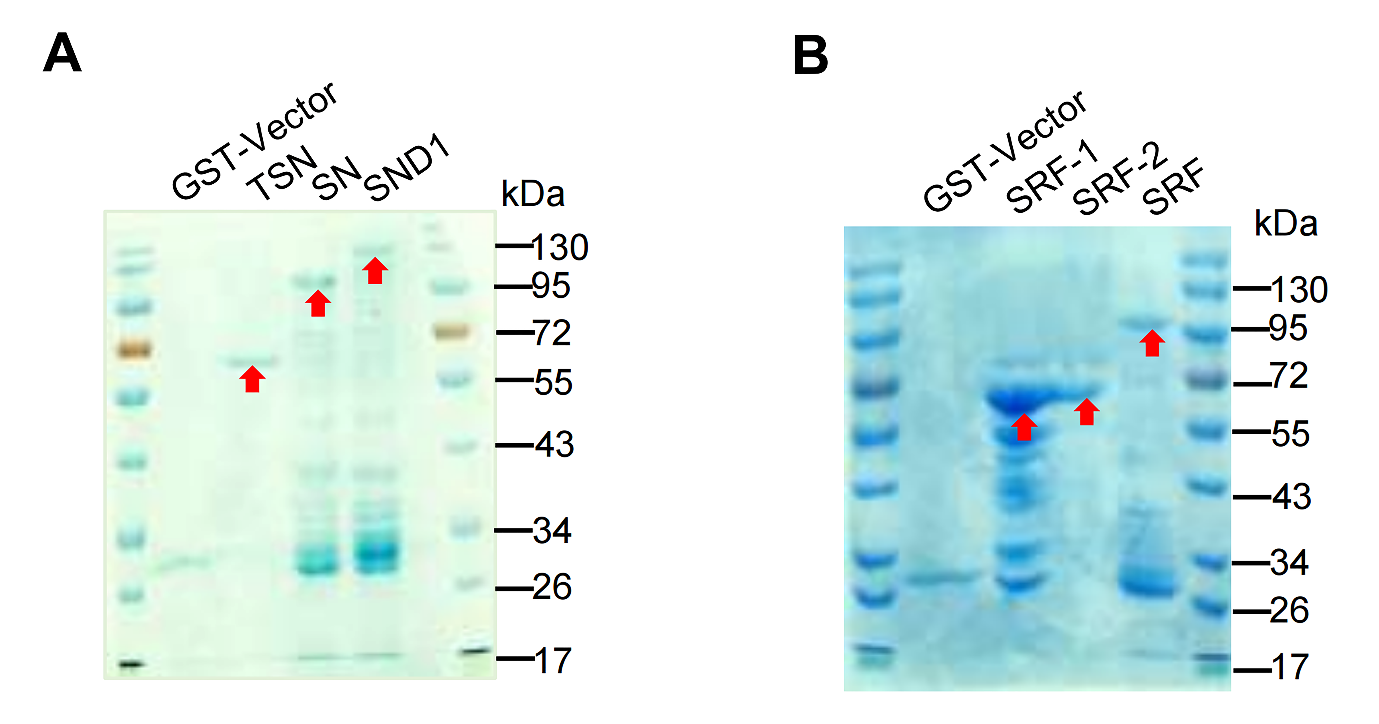


**Fig. S5** **Coomassie blue staining for GST-fusion proteins. (A)** Coomassie blue staining for GST-fusion proteins matched Fig. 5C. **(B)** Coomassie blue staining for GST fusion proteins matched Fig. 5D.

**
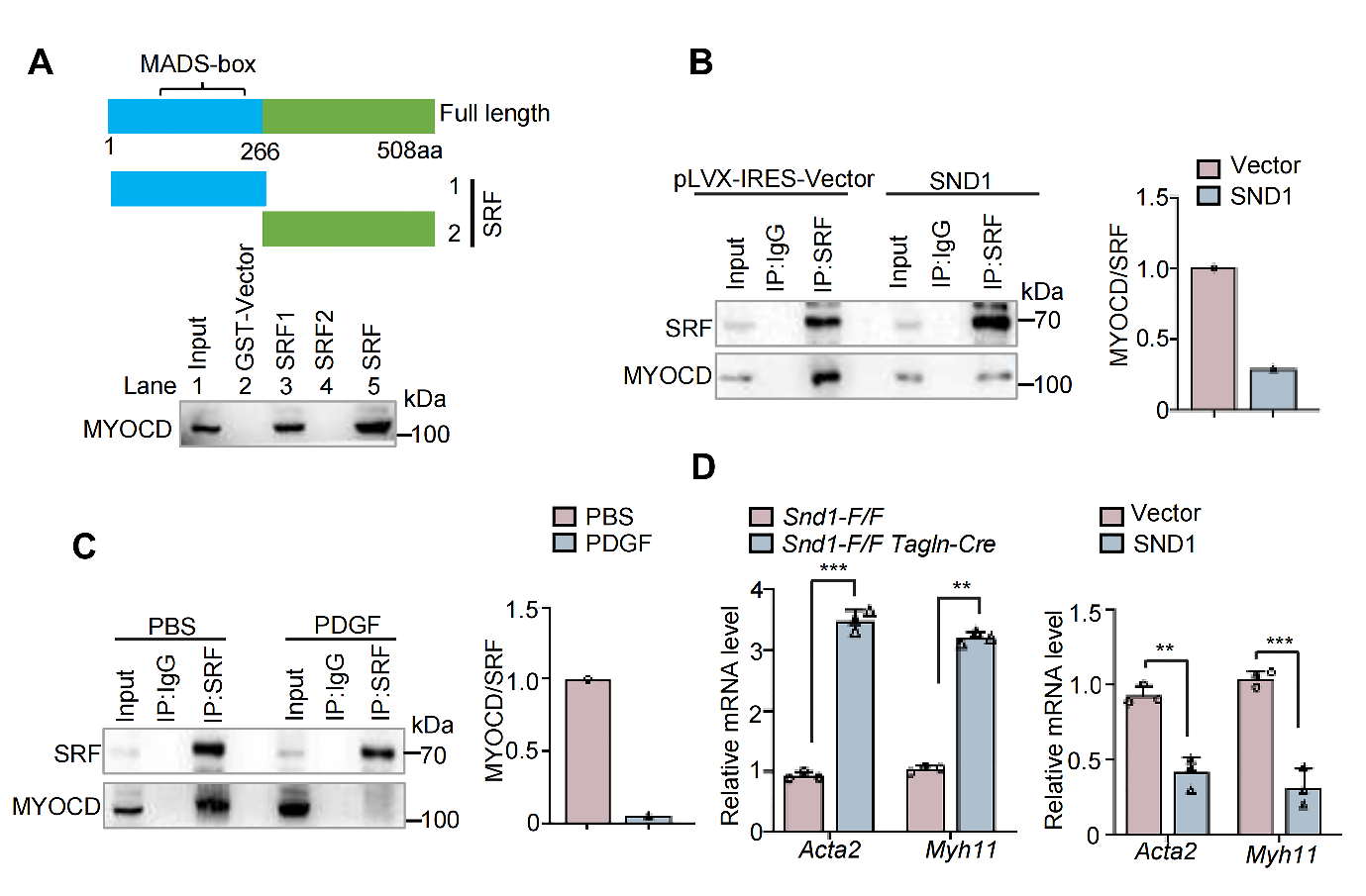
**

**Fig. S6 SND1 competes with MYOCD to interact with SRF. (A)** GST pull-down assay analyses the interaction of GST-fusion protein containing full-length SRF (GST-SRF), SRF-1 domain (containing MADS box), and SRF-2 domain with His-MYOCD. **(B)** The primary VSMCs were infected with pLVX-IRES-SND1 or pLVX-IRES-Vector.1.5 ×10^7^ cells were collected for Co-IP assay with IgG or SRF antibody. **(C)** The primary VSMCs were treated with PDGF (40 ng/mL) for 24 h after being cultured in serum-free DMEM for 24 h, 1.5 ×10^7^ cells were collected for Co-IP assay with IgG or SRF antibody. The immunoprecipitated complex was immunoblotted with SRF and MYOCD antibodies. **(D)** Primary VSMCs from *Snd1-F/F* and *Snd1-F/F Tagln-Cre* mice were isolated and cultured. 2×10^6^ primary VSMCs were obtained in 6 cm dish. The primary VSMCs were infected with pLVX-IRES-SND1 or pLVX-IRES-Vector. The mRNA levels of *Acta2* and *Myh11* were detected by RT-qPCR. Western blotting results were analyzed by ImageJ (2×) software. Statistical analysis was performed by unpaired two-tailed Student’s t-test. ***P* < 0.01, ****P* < 0.001.


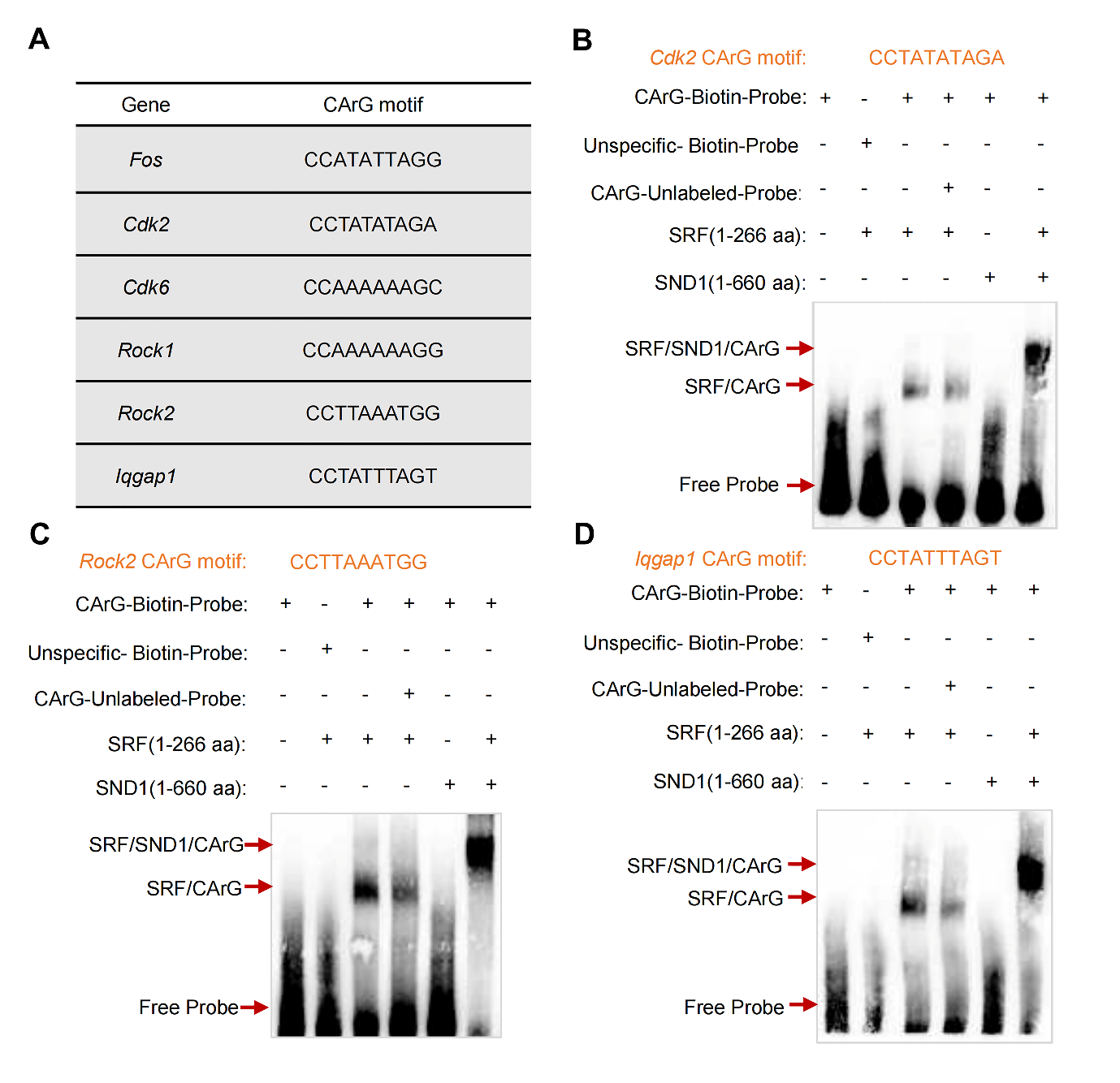


**Fig. S7 SND1 bound with SRF-CArG complex. (A)** The CArG motif in promoter region of the SRF-related proliferation and migration genes**. (B-D)** EMSA assays were performed using SRF1(1–266 aa, 0.1 μg), SND1 (1–660 aa, 0.1 μg) purified proteins and Biotin-labeled/unlabeled probes targeting the CArG motif in the *Cdk2*, *Rock2*, and *Iqgap1* promoters.


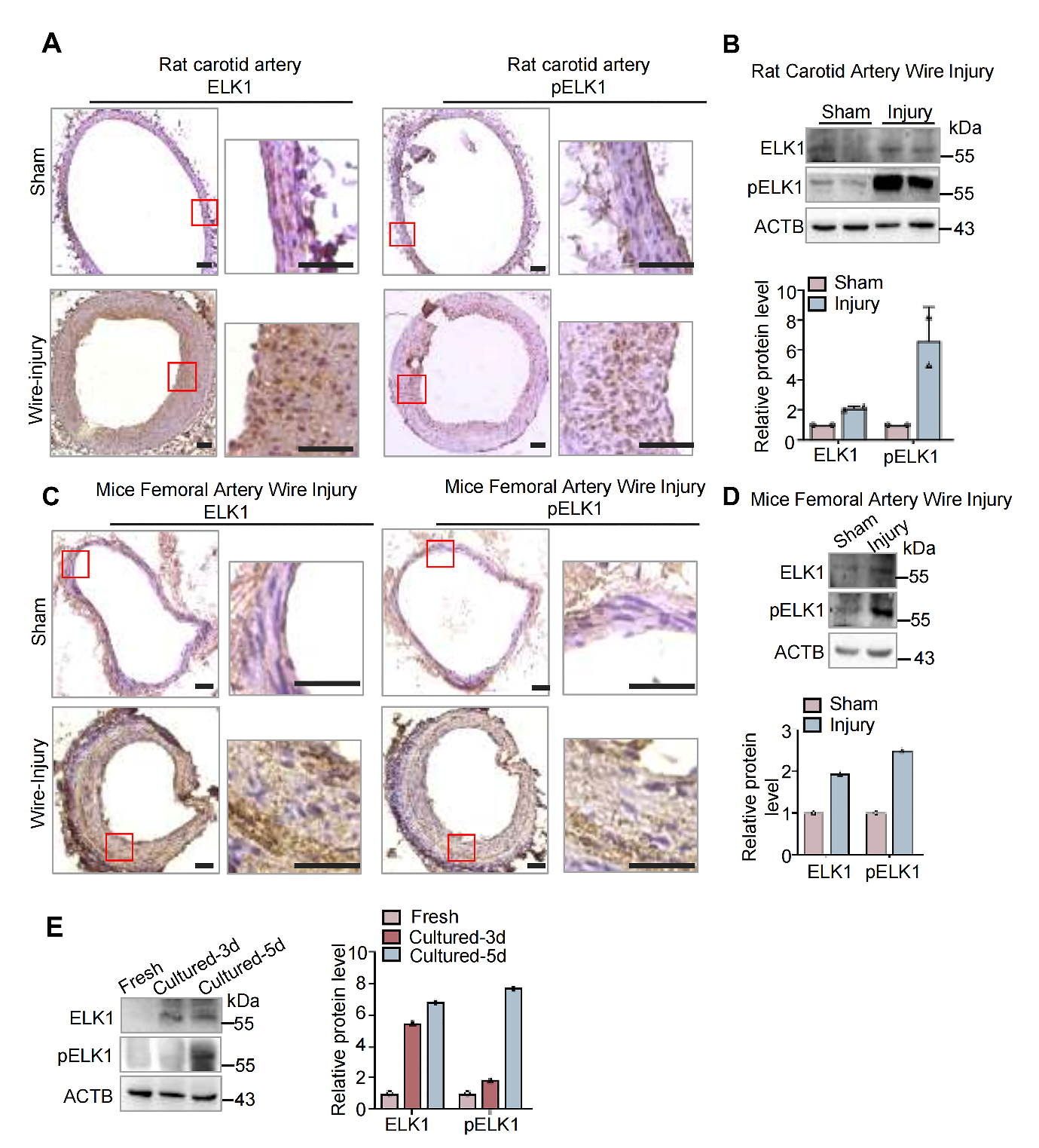


**Fig. S8 Vascular injury increased the expression of** **ELK1 and pELK1.** Wire-induced rat carotid artery injury (n = 6 /group) and mouse femoral artery injury) models (n = 6 /group) were generated. The carotid and femoral arteries were collected at 14 or 28 days after the injury, respectively. **(A, C)** Immunohistochemical staining of ELK1 and pELK1. **(B, D)** The protein levels of ELK1, pELK1 and ACTB were detected by western blotting. **(E)** Mice thoracic aortas were isolated and collected (Fresh) or cultured for 3 days or 5 days. The protein levels of ELK1, pELK1 and ACTB were detected by western blotting. All western blotting results were analyzed by ImageJ (2×) software. Scale bar, 200 μm in A; 100 μm in C.


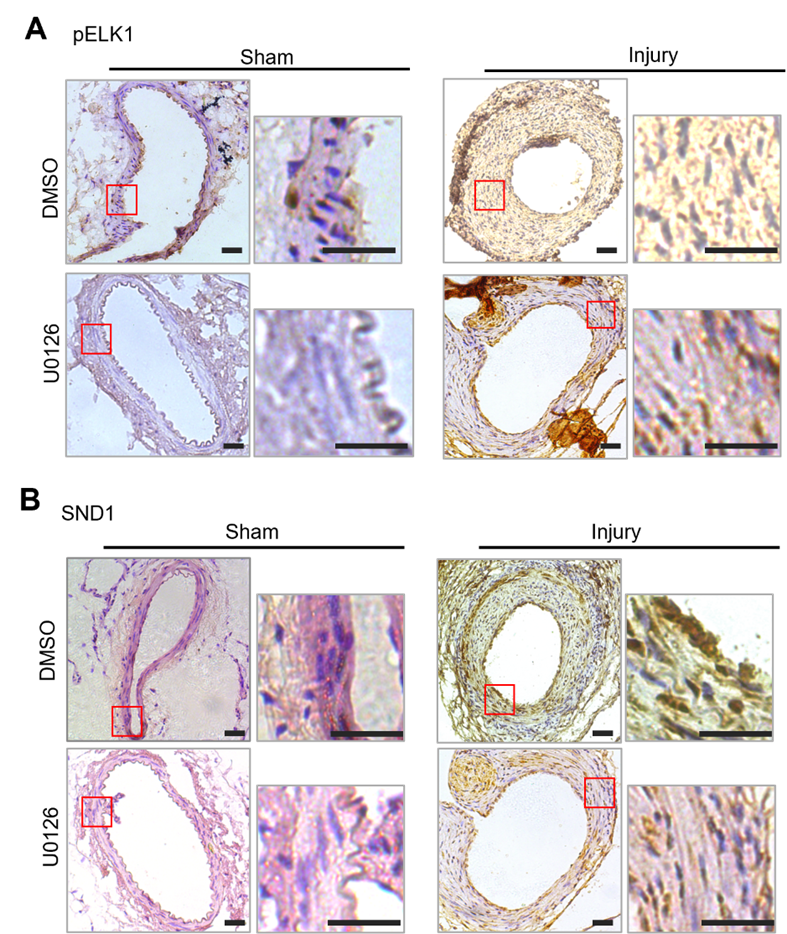


**Fig. S9 The phosphorylation of ELK1 and the upregulation of SND1 in the injured femoral arteries were inhibited by U0126 treatment.** Wire-induced mouse femoral artery injury models were constructed, and U0126 was injected intraperitoneally every 3 days (1 mg/kg) after wire injury, DMSO as the control. The femoral arteries were collected (n = 4/group). **(A)** Immunohistochemical staining of pELK1. **(B)** Immunohistochemical staining of SND1. Scale bar, 100 μm.


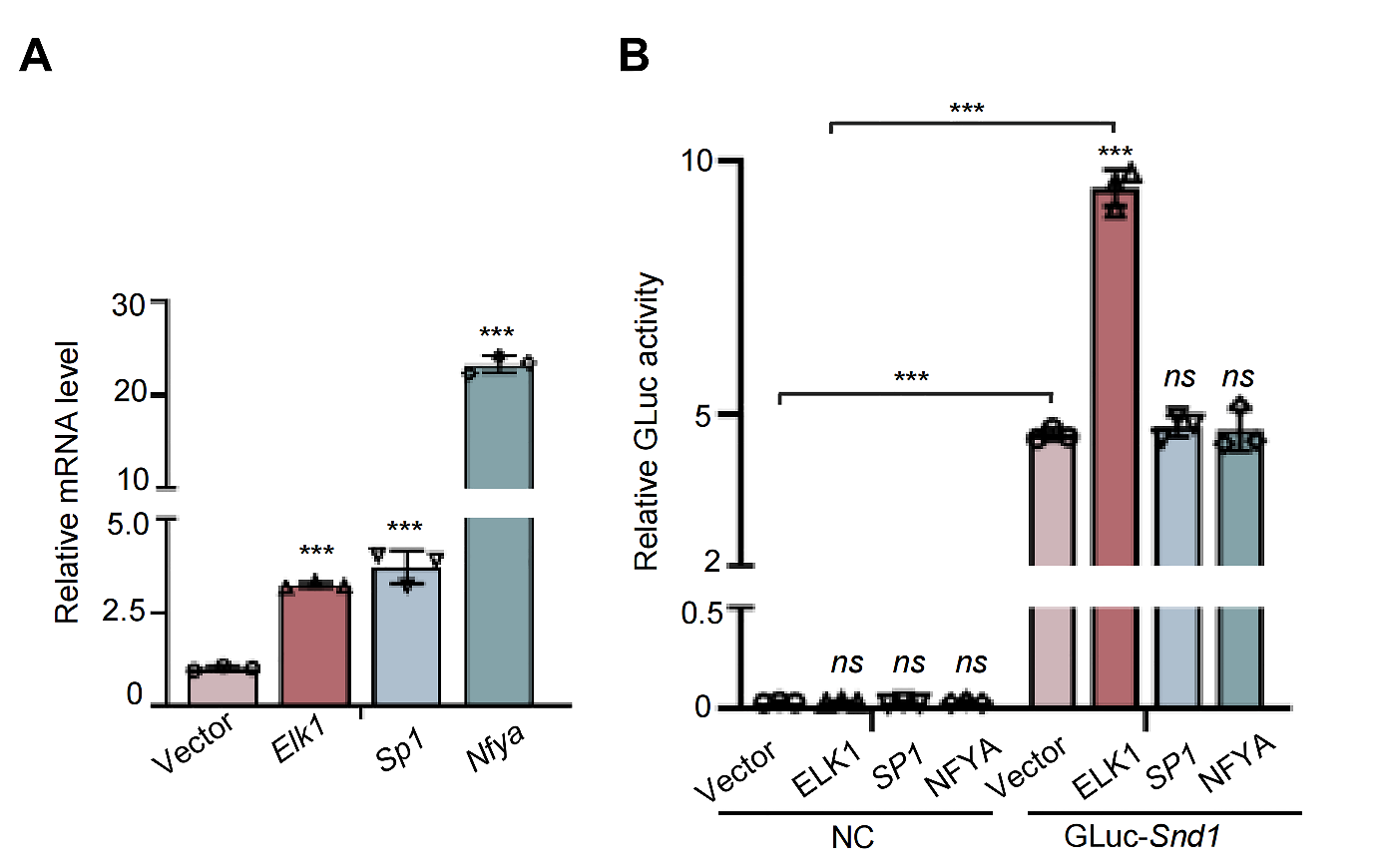


**Fig. S10 Transcription factors SP1 and NFYA did not influence the transcriptional activity of *Snd1* in the VSMCs. (A)** The primary VSMCs were infected with pLVX-IRES-Vector, pLVX-IRES-ELK1, pLVX-IRES-SP1, or pLVX-IRES-NFYA (2×10^5^ per well in 6-well plates). RT-qPCR showed the infection efficiency of transcription factors plasmids (*Elk1, Sp1, and Nfya*). **(B)** The primary VSMCs were infected with the empty GLuc-Vector (NC) or GLuc-*Snd1* promoter plasmids, together with transcription factor plasmids (ELK1, SP1, and NFYA) or the pLVX-IRES-Vector (2×10^5^ per well in 6-well plates). The relative luciferase activity was evaluated by the ratio of GLuc / SEAP activity. All data are present as mean ± SD (n = 3). Statistical analysis was performed by unpaired two-tailed Student’s t-test (A) and two-way ANOVA (B). ****P* < 0.001; *ns*, no significance.


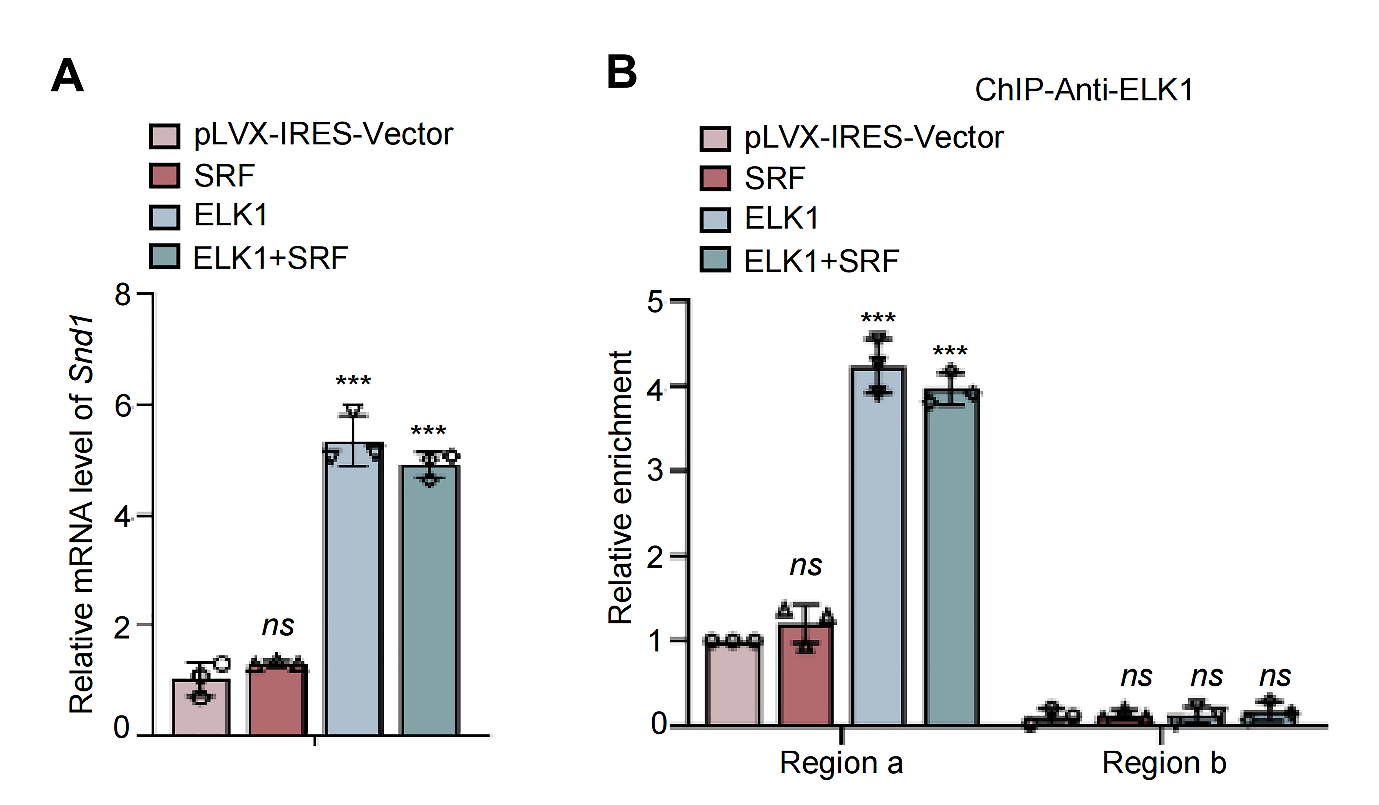


**Fig. S11** **ELK1 regulated *Snd1* transcription without collaborating with SRF**. The primary VSMCs were infected with pLVX-IRES-Vector, pLVX-IRES-SRF, pLVX-IRES-ELK1*,* or pLVX-IRES-SRF plus ELK1 (2×10^6^ per well in 6 cm dish)*.* **(A)** The mRNA level of the *Snd1* was detected by RT-qPCR, and **(B)** ELK1 enrichment at different regions of *Snd1* promoter was detected by ChIP. All data are present as mean ± SD (n = 3). Statistical analysis was performed with one-way ANOVA. ****P* < 0.001, *ns*: no significance.

**Supplemental Tables**

**Table S1 Primers for genotyping**

| **Gene** | **Primer** |
| --- | --- |
| Flox- Forward primer | CAGCACTAAAAGCTTGTCCC |
| Flox- Reverse primer | GCTAAAGAGTCCCTAGAAAG |
| *Tagln*-*Cre*- Forward primer | CTCCTTCCAGTCCACAAACGACC |
| *Tagln*-*Cre*- Reverse primer | GGCGATCCCTGAACATGTCC |

**Table S2 Sequences of shRNA**

| **Gene** | **Primer** |
| --- | --- |
| shElk1-1- Forward primer | CCGGGCAGTTTCTGCTGCAGCTTCTCTCGAGAGAAGCTGCAGCAGAAACTGCTTTTTG |
| shElk1-1- Reverse primer | AATTCAAAAAGCAGTTTCTGCTGCAGCTTCTCTCGAGAGAAGCTGCAGCAGAAACTGC |
| shElk1-2- Forward primer | CCGGGCAGCTTCTGAGAGAACAAGGCTCGAGCCTTGTTCTCTCAGAAGCTGCTTTTTG |
| shElk1-2- Reverse primer | AATTCAAAAAGCAGCTTCTGAGAGAACAAGGCTCGAGCCTTGTTCTCTCAGAAGCTGC |

**Table S3 Primers for real-time PCR analysis**

| **Gene** | **Primer** |
| --- | --- |
| Snd1- Forward primer | TGTGCCACTGTCACCATTGGAG |
| Snd1- Reverse primer | CAGCTCATCGTAGTGTGAAGACC |
| Actb- Forward primer | CATTGCTGACAGGATGCAGAAGG |
| Actb - Reverse primer | TGCTGGAAGGTGGACAGTGAGG |
| Elk1- Forward primer | GGAACAAGCTCTGGTCTTCAGG |
| Elk1- Reverse primer | CTATGCAGGCTGTGGCAAAACC |
| Sp1- Forward primer | CTCCAGACCATTAACCTCAGTGC |
| Sp1- Reverse primer | CACCACCAGATCCATGAAGACC |
| Nfya- Forward primer | GGAATGTGGTCAACTCAGGAGG |
| Nfya- Reverse primer | CTTCTTCCAGCATCTCTGCTCC |
| Cdk2- Forward primer | TCATGGATGCCTCTGCTCTCAC |
| Cdk2- Reverse primer | TGAAGGACACGGTGAGAATGGC |
| Cdk6- Forward primer | ACCTCTGGAGTGTCGGTTGCAT |
| Cdk6- Reverse primer | TTCCTCTCCTGGGAGTCCAATG |
| Fos- Forward primer | GGGAATGGTGAAGACCGTGTCA |
| Fos- Reverse primer | GCAGCCATCTTATTCCGTTCCC |
| Rock1- Forward primer | CACGCCTAACTGACAAGCACCA |
| Rock1- Reverse primer | CAGGTCAACATCTAGCATGGAAC |
| Rock2- Forward primer | GTGACCTCAAACAGTCTCAGCAG |
| Rock2- Reverse primer | GACAACGCTTCTGAGTTTCCTGC |
| Iqgap1- Forward primer | ACCAGAGTGACCTTGCTGAAGC |
| Iqgap1- Reverse primer | TTGCGTCTCCAGGTTGTGGTAG |
| Acta2- Forward primer | TGCTGACAGAGGCACCACTGAA |
| Acta2- Reverse primer | CAGTTGTACGTCCAGAGGCATAG |
| Myh11- Forward primer | GCAACTACAGGCTGAGAGGAAG |
| Myh11- Reverse primer | TCAGCCGTGACCTTCTCTAGCT |

**Table S4 Primers for ChIP analysis**

| **Gene** | **Primer** |
| --- | --- |
| Snd1-Regain a- Forward primer | GGAGCAGAACCTCCGCTGGA |
| Snd1-Regain a- Reverse primer | CGCTGAAAGAGCTGATTTGC |
| Snd1-Regain b- Forward primer | TTGGCTTGAGTTCCAGCGGG |
| Snd1-Regain b- Reverse primer | GCGACAGGCTACCGTCGGTG |
| Cdk2-promoter region- Forward primer | ACACTCACCTTCTAGGGCC |
| Cdk2-promoter region- Reverse primer | AGAAACTGTCATTGAGCTGT |
| Fos-promoter region- Forward primer | GAATGTTCGCTCGCCTTCT |
| Fos-promoter region- Reverse primer | TTAGGGGGTCTCCTAGACC |
| Cdk6-promoter region- Forward primer | TATTCAAAGACAGCTATTT |
| Cdk6-promoter region- Reverse primer | CACCAAGGTATTTCTGGAG |
| Iqgap1-promoter region- Forward primer | CTAGATGCTTCAGAAATAG |
| Iqgap1-promoter region- Reverse primer | GACTCTTACCCTCACCATAA |
| Rock1-promoter region- Forward primer | AGGCCAGCCTGGTCTAGAG |
| Rock1-promoter region- Reverse primer | GCACAGATTTAAAGGATTCC |
| Rock2-promoter region- Forward primer | AGAGCTTGTTTGGATTATT |
| Rock2-promoter region- Reverse primer | CTAGCCATTGGTCAGATCC |
| Cdk2-negative region-Forward primer | TCACCGTGTCCTTCACCGAG |
| Cdk2-negative region-Reverse primer | TGCAGCCCAGGCTCCAGATAT |
| Cdk6-negative region-Forward primer | CCACAGAAACCATAAAGGAT |
| Cdk6- negative region-Reverse primer | CTTCTGGGGCTCGGTACCAC |
| Fos-negative region-Forward primer | AGCGGAGACAGATCAACTTG |
| Fos-negative region-Reverse primer | TCCTCAGACTCTGGGGTGGA |
| Rock1-negative region-Forward primer | CAGAAGATTATGAAGTGGTA |
| Rock1-negative region-Reverse primer | CTTGGAATGCATAAAAAAGCT |
| Rock2-negative region-Forward primer | ATGGCCTTTGCCAACAGTCC |
| Rock2-negative region-Reverse primer | TCACATCTCTGTGTATTAAG |
| Iqgap1-negative region-Forward primer | AATCTATGATCGAGAACAGA |
| Iqgap1-negative region-Reverse primer | AGGAGCCAGGCCCAGTTTGA |

**Table S5 Primers for EMSA**

| **Gene** | **Probe** |
| --- | --- |
| Fos- Forward primer | CCATATTAGGCCATATTAGGCCATATTAGGCCATATTAGGCCATATTAGG |
| Fos- Reverse primer | CCTAATATGGCCTAATATGGCCTAATATGGCCTAATATGGCCTAATATGG |
| Cdk2- Forward primer | CCTATATAGACCTATATAGACCTATATAGACCTATATAGACCTATATAGA |
| Cdk2- Reverse primer | TCTATATAGGTCTATATAGGTCTATATAGGTCTATATAGGTCTATATAGG |
| Rock2-Forward primer | CCTTAAATGGCCTTAAATGGCCTTAAATGGCCTTAAATGGCCTTAAATGG |
| Rock2- Reverse primer | CCATTTAAGGCCATTTAAGGCCATTTAAGGCCATTTAAGGCCATTTAAGG |
| Iqgap1- Forward primer | CCTATTTAGTCCTATTTAGTCCTATTTAGTCCTATTTAGT CCTATTTAGT |
| Iqgap1- Reverse primer | ACTAAATAGGACTAAATAGGACTAAATAGGACTAAATAGGACTAAATAGG |
| SND1-Forward primer | CCGGAAGTCCGGAAGTCCGGAAGTCCGGAAGTCCGGAAGT |
| SND1- Reverse primer | ACTTCCGGACTTCCGGACTTCCGGACTTCCGGACTTCCGG |

**Table S6 Antibody information**

| **Antibody** | **Corporation** | **Catalog** | **Working concentration** |
| --- | --- | --- | --- |
| anti-ACTA2 | abcam | ab7817 | WB: 1:200;  IHC-P/ IF:1μg/ml |
| anti-SND1 | abcam | ab65078 | WB: 1μg/ml; IHC-P:5μg/ml |
| anti-MYH11 | abcam | ab683 | WB: 1:1000; |
| anti-ELK1 | abcam | ab32106 | ChIP: 1:50-1:100; IHC-P: 1:50 |
| anti-ELK1 | SAB | 48811 | WB: 1:1000; |
| anti-pELK1 | SAB | 11004 | WB: 1:500; IHC-P: 1:50 |
| anti-MYOCD | SAB | 44757 | WB：1:2000 |
| anti-Calponin (CNN1) | proteintech | 24855-1-AP | WB: 1:1000 |
| anti-PCNA | proteintech | 10205-2-AP | WB: 1:2000;  IF: 1:50 |
| anti-FOS | proteintech | 66590-1-lg | WB: 1:5000 |
| anti-SRF | proteintech | 16821-1-AP | WB: 1:1000;  IP: 2-4μg  ChIP: 2-4μg |
| anti-ROCK1 | proteintech | 21850-1-AP | WB: 1:2000 |
| anti-ROCK2 | proteintech | 21645-1-AP | WB: 1:2000 |
| anti-IQGAP1 | proteintech | 22167-1-AP | WB: 1:2000 |
| anti-CDK6 | CST | 13331 | WB: 1:1000 |
| anti-CDK2 | CST | 18048 | WB: 1:1000 |
| anti-GCN5L2 (KAT2B) | CST | 3305 | WB: 1:1000  IP: 1:200 |
| anti-Acetyl-Histone H3(Lys9) | CST | 9649 | ChIP: 1:50 |
| anti-Acetyl-Histone H3 (Lys27) | CST | 8173 | ChIP: 1:100 |
| anti-Ki-67 | Invitrogen | PA5-19462 | IHC:1μg/ml |
| anti-ACTB | Sigma Aldrich | A5441 | WB: 1:5000 |
| Normal Rabbit IgG Polyclonal Antibody | Sigma Aldrich | 12-370 | IP: 2 µg |
| Normal Mouse IgG Polyclonal Antibody | Sigma Aldrich | 12-371 | IP: 2 µg |
| Peroxidase-labeled-goat anti-mouse IgG | KPL | 074-1806 | WB: 1:5000 |
| Peroxidase-labeled-goat anti-Rabbit IgG | KPL | 074-1506 | WB:1:5000 |
| Donkey anti-Rabbit IgG (H+L) Highly Cross-Adsorbed Secondary Antibody, Alexa Fluor™ 546 | Thermo Fisher | A10040 | IF:4 µg/mL |
| Goat anti-Mouse IgG (H+L) Cross-Adsorbed Secondary Antibody, Alexa Fluor™ 488 | Thermo Fisher | A-11001 | IF:1 µg/mL |
